# Cost-effectiveness of strength exercise or aerobic exercise compared with usual care for patients with knee osteoarthritis: secondary results from a multiarm randomised controlled trial in Norway

**DOI:** 10.1136/bmjopen-2023-079704

**Published:** 2024-05-23

**Authors:** Rikke Munk Killingmo, Britt Elin Øiestad, May-Arna Risberg, Esther Maas, Margreth Grotle

**Affiliations:** 1 Department of Rehabilitation Science and Health Technology, Oslo Metropolitan University, Oslo, Norway; 2 Division of Orthopaedic Surgery, Oslo Universitetssykehus, Oslo, Norway; 3 Department of Sports Medicine, Norwegian School of Sports Sciences, Oslo, Norway; 4 Department of Health Sciences, Vrije University Amsterdam, Amsterdam, The Netherlands; 5 The Amsterdam Movement Sciences Research Institute, Amsterdam, The Netherlands; 6 Research and Communication Unit for Musculoskeletal Health, Oslo universitetssykehus Ulleval, Oslo, Norway

**Keywords:** health economics, physical therapy modalities, primary health care

## Abstract

**Objectives:**

To evaluate the 1-year cost-effectiveness of strength exercise or aerobic exercise compared with usual care for patients with symptomatic knee osteoarthritis (OA), from a societal and healthcare perspective.

**Design:**

Cost-effectiveness analysis embedded in a three-arm randomised controlled trial.

**Participants and setting:**

A total of 161 people with symptomatic knee OA seeking Norwegian primary or secondary care were included in the analyses.

**Interventions:**

Participants were randomised to either 12 weeks of strength exercise (n=54), 12 weeks of aerobic exercise (n=53) or usual care (n=54).

**Outcome measures:**

Quality-adjusted life-years (QALYs) estimated by the EuroQol-5 Dimensions-5 Levels, and costs related to healthcare utilisation and productivity loss estimated in euros (€), aggregated for 1 year of follow-up. Cost-effectiveness was expressed with mean incremental cost-effectiveness ratios (ICERs). Bootstrapping was used to estimate ICER uncertainty.

**Results:**

From a 1-year societal perspective, the mean cost per patient was €7954, €8101 and €17 398 in the strength exercise, aerobic exercise and usual care group, respectively. From a 1-year healthcare perspective, the mean cost per patient was €848, €2003 and €1654 in the strength exercise, aerobic exercise and usual care group, respectively. Mean differences in costs significantly favoured strength exercise and aerobic exercise from a 1-year societal perspective and strength exercise from a 1-year healthcare perspective. There were no significant differences in mean QALYs between groups. From a 1-year societal perspective, at a willingness-to-pay threshold of €27 500, the probability of strength exercise or aerobic exercise being cost-effective was ≥98%. From a 1-year healthcare perspective, the probability of strength exercise or aerobic exercise being cost-effective was ≥97% and ≥76%, respectively.

**Conclusion:**

From a 1-year societal and healthcare perspective, a 12-week strength exercise or aerobic exercise programme is cost-effective compared with usual care in patients with symptomatic knee OA.

**Trial registration number:**

NCT01682980.

**STRENGTHS AND LIMITATIONS OF THIS STUDY**

- The main strength of the present study is that it was a cost-effectiveness study of a three-arm randomised controlled trial (RCT), that it was conducted in line with the Panel on Cost-Effectiveness in Health and Medicine guidelines, and preplanned with a published statistical analysis plan. Furthermore, that bootstrapping methods were used to determine uncertainty around calculated incremental cost-effectiveness ratios.
- The main limitation of the present study is that it was powered on the primary outcome of the three-arm RCT, not on the cost-effectiveness outcome. However, this is common practice and bootstrapped estimates for result interpretation were used.
- The present study excluded people >70 years of age, and it is unknown whether results are generalisable to this subgroup of people with knee osteoarthritis. Costs related to productivity loss are expected to be markedly lower among people >70 years. Moreover, people with self-reported body mass index >35 kg/m^2^ were excluded.
- Due to between-country differences in healthcare organisations and mechanisms of compensation for productivity loss, readers are advised to be cautious with the generalisation of results to other healthcare systems.

## Introduction

 The burden of knee osteoarthritis (OA) has been growing along with an increasing and ageing population.^1 2^ Knee OA imposes a leading cause of disability globally,^1^,^4^ and an extensive burden to society with high costs related to both healthcare utilisation and productivity loss.^14^,^6^ Clinical guidelines for the management of knee OA consistently recommend patient education, exercise and weight management (if appropriate) as core treatments, regardless of disease severity and comorbidity.^7^,^9^ Core treatments have been stated to be safe, appropriate and effective, yet uptake of these proven treatments seems to be low in clinical practice.^10 11^ One approach to support implementation of core treatments in healthcare systems and reimbursement plans is to evaluate cost-effectiveness (CE).^11^,^13^ CE analyses (CEAs) explicitly quantify the relative costs and benefits of different interventions. Thus, CEAs aim to illuminate potential trade-offs and inform discussions on resource allocation in healthcare.^6 12^ The evidence base for CE of knee OA core treatments is still relatively limited,^11 14^ and researchers have highlighted that further knowledge is needed to bolster implementation of appropriate healthcare plans, improve the use of scarce healthcare resources and reduce the global burden of knee OA.^6 11 15^

A three-arm randomised controlled trial (RCT)^16^ evaluated the effectiveness of a 12-week strength exercise (SE) or aerobic exercise (AE) programme, compared with usual care (UC), on knee-related quality of life after 1 year in individuals with symptomatic knee OA. The trial found no statistically significant difference in effects between groups. A result generally in accordance with previous research^17^ showing a gradual decrease in exercise effects over time, being no better than UC at around 9 months. However, the lack of statistical differences in effects does not necessarily mean that interventions are identical in terms of CE. Therefore, the Professional Society for Health Economics and Outcomes Research Cost-Effectiveness Analysis Alongside Clinical Trials (RCT-CEA) Task Force^18^ recommend researchers to perform CEAs regardless of clinical results. Furthermore, it has been recommended to conduct these analyses preferably from both a societal and healthcare perspective.^612 18^,^20^

Therefore, the aim of this study was to evaluate the 1-year CE of SE or AE compared with UC for patients with symptomatic knee OA, from a societal and healthcare perspective.

## Method

The method of this study has been reported previously in the study protocol^21 22^ and in the paper on clinical effectiveness.^16^ For the CE part of the study, the reference case for economic evaluations in OA,^6^ the Panel on Cost-Effectiveness in Health and Medicine guidelines,^19 20^ as well as the Consolidated Health Economic Evaluation Reporting Standards (CHEERS) Statement^23^ were used. A summary of the method is presented below.

### Design and setting

This study is a CEA embedded in a three-arm RCT, evaluating the effect of SE or AE, compared with UC, on knee-related quality of life in individuals with symptomatic knee OA. The trial had a treatment phase of 2 plus 12 weeks and a follow-up phase of 12 months.

### Participants, recruitment procedure and randomisation

Eligible participants were people with symptomatic knee OA (confirmed mild to moderate radiographic, Kellgren and Lawrence^24^ grades 2–3), aged 35–70 years, who fulfilled 3/4 of the American College of Rheumatology clinical criteria (stiffness <30 min, crepitus, osteophytes, pain last days last month).^25^ Excluded were those with serious physical or mental illness, serious musculoskeletal impairment in the lower extremities or low back, lower extremity prosthesis, self-reported body mass index >35 kg/m^2^, scheduled surgery within 6 months, Norwegian language barriers and those already participating in structured, weekly, SE or AE. Participants were recruited from primary and secondary care in Norway between April 2013 and March 2020 (the main reasons for a slow recruitment rate were a lack of time and few resources available to recruit on a weekly basis). All included participants signed an informed consent before study enrolment. Participants were randomised to either SE, AE or UC with a 1:1:1 ratio within block sizes of 6. A biostatistician, not involved in the project, prepared the computer-generated randomisation lists. Group allocation was concealed for the researchers and the self-reported data assessors.

### Interventions

A detailed description of the rationale, development and content of the interventions can be found elsewhere.^16^ Briefly, the SE group was followed up closely by an experienced physiotherapist, received an individual, supervised programme based on a previously developed exercise programme for knee patients (including neuromuscular and SEs)^26^ and was told to exercise 2–3 times a week for 12 weeks plus a 2-week preparation phase to be familiar with the programme and optimise exercise techniques. The AE group received a stationary cycling programme based on guidelines for aerobic training parameters in people with pain associated with OA (10 min warm up, 30 min on moderate intensity (75% of maximum heart rate) and 5 min on low intensity)^27^ and was told to exercise 2–3 times a week for 12 weeks plus a 2-week preparation phase. The number of physiotherapy consultations included in the SE and AE intervention was flexible and individually determined. A minimum of two exercise sessions per week were supervised by the physiotherapist while the third session could be performed unsupervised at home. All supervised SE and AE exercise sessions took place at physiotherapy clinics situated near the participant’s homes. All participants exercised individually. The UC group was told to avoid starting a new exercise programme involving structured SE or AE until the 4 months of follow-up was completed but otherwise live as usual.

### Data collection, effect and cost measures

At baseline (before random group allocation), all participants completed a comprehensive questionnaire. Follow-up questionnaires covering healthcare utilisation, productivity loss (sick leave, work assessment allowance and disability benefits) and health state were sent at 14 weeks (postintervention) and 12 months after inclusion. All questionnaires were completed on paper. In addition, healthcare utilisation and productivity loss were also recorded at 6 and 9 months after inclusion by phone interviews. Furthermore, public registry data on knee replacement surgery was collected from the Norwegian Arthroplasty Register, in the period from baseline to 12 months follow-up.

#### Effect measure

The effect measure of this study was health-related quality of life expressed by quality-adjusted life-years (QALYs). First, health state of the participants was measured by the EuroQol-5 Dimensions-5 Levels (EQ-5D-5L).^28^ Second, the UK tariff^29^ was used to convent health states into utility scores (range −0.59 to 1), anchored at 0 ‘death’ and 1 ‘perfect health’, with negative values representing health states perceived to be worse than death. The UK tariff was used, as a Norwegian tariff is not available. QALYs were estimated as area under the curve using the trapezoidal method.^30^ QALYs remain undiscounted due to follow-up being confined to 1 year.^30^

#### Cost measures

This study adopted a societal and healthcare perspective, thus both costs related to healthcare utilisation and productivity loss were included. Healthcare utilisation due to knee OA (including physiotherapist consultations related to the study interventions) was self-reported and included consultations with healthcare professionals (type and frequency) (online supplemental additional file 1, figure A1). In addition, information on knee replacement surgery was obtained from the Norwegian Arthroplasty Register. Productivity loss due to knee OA was self-reported and included the number of calendar days with sick leave, work assessment allowance and disability benefits (online supplemental additional file 1, figure A1). Workers in Norway qualify for sickness benefits from the public welfare agency if they have been in paid work for minimum 4 weeks before the sickness incident, and if the occupational disability is documented by a doctor’s sick leave certificate. In general, sickness benefits can be received from the first day of reported sick and up to 1 year. If a person is still unable to work after 1 year, he or she may be entitled to work assessment allowance or disability benefits.

Healthcare utilisation and productivity loss were reported with a 3-month recall period at each time point of follow-up. A number of calendar days with productivity loss were converted into workdays with productivity loss and adjusted for employment rate as well as reported grading of productivity loss, summarising the number of workdays with part-time productivity loss to the number of workdays with complete productivity loss.

### Analyses

All analyses were preplanned in a statistical analysis plan published a priori^31^ and performed using the IBM SPSS V.26 (IBM) or Stata version 16.1 (StataCorp). The intention-to-treat (ITT) method was used. P values <0.05 were considered statistically significant. All statistical tests were two sided.

#### Study flow

Flow of participants through the study was reported according to the Consolidated Standards of Reporting Trials Statement^32^ with a flow chart. Baseline differences between responders (at all time points) and non-responders (at minimum one follow-up time point) were evaluated through frequency and percentage.

#### Missing data

Missing value patterns were visually explored, and missingness at random was assumed. Also, we found evidence against the hypothesis that values were not missing completely at random (Little’s test, p>0.05). Missing data were handled by multiple imputation. Five multiple imputation datasets with 10 iterations were created using regression estimation, and pooled estimates were calculated using Rubin’s rules.^33^

#### Healthcare utilisation, productivity loss and cost estimation

Type and frequency of use of different healthcare resources (including physiotherapist consultations related to the study interventions) and type and frequency of productivity loss were calculated for each of the follow-up periods. Costs of healthcare utilisation per patient were estimated by multiplying frequency of use by unit costs collected from national pricelists (table 1). Non-healthcare costs related to the provision of healthcare (such as transportation and exercise equipment) were not estimated. Costs of productivity loss per patient were estimated by multiplying number of workdays with complete productivity loss by an estimated average wage rate including taxes and social costs (table 1). All costs were presented in euros (€) 2020 and estimated with mean (SD) values for the three groups, for the following follow-up periods: 0 to 3 months, >3 to 12 months and 0 to 12 months. Costs remain undiscounted due to follow-up being confined to 1 year.^30^ Norwegian prices were recalculated to euros using the exchange rate from the Norwegian Bank of Norway from February 2020 (€1=NOK10).

**Table 1:** Cost categories, units, unit price, all numbers in euros (€) and Norwegian kroner (NOK) for 2020

Cost categories	Unit	Unit price (€)	Unit price (NOK)	Reference (source)
Primary care				
General practitioner	Per visit	43.1	431	The Norwegian Medical Association, estimated average
Physiotherapist	Per visit	47.2	472	The Norwegian Physiotherapy Association, estimated average
Chiropractor	Per visit	55.0	550	Private price lists, estimated average
Manuel therapist	Per visit	74.2	742	The Norwegian Physiotherapy Association, estimated average
Psychomotoric physiotherapist	Per visit	74.2	742	The Norwegian Physiotherapy Association, estimated average
Other therapists	Per visit	75.0	750	Private price lists, estimated average
Secondary care				
Medical specialist	Per visit	90.0	900	The Norwegian Medical Association, estimated average
Knee replacement surgery	Per surgery	9248.6	92 486	DRG 209H
Productivity loss				
Sick leave (252 workdays per year)	Per workday	357.2	3572	Statistics Norway, estimated average including taxes/social costs
Work assessment allowance	Per workday	235.8	2358	Statistics Norway, estimated average including taxes/social costs
Disability benefits	Per workday	235.8	2358	Statistics Norway, estimated average including taxes/social costs

#### CE analysis

Differences between groups in costs (SE or AE–UC) were described with means (95% CI) and evaluated with Student’s t-test. Differences between groups in QALYs (SE or AE–UC) were described with means (95% CI) and evaluated with ANCOVA to adjust for baseline scores unequally distributed across the three groups at baseline (sex, occupational status, EQ-5D-5L score and healthcare utilisation prior to inclusion). CE was estimated with mean incremental cost-effectiveness ratios (ICERs) by dividing mean differences in costs by mean differences in effects. The CE threshold (willingness-to-pay, WTP) for OA was based on the Norwegian governmental report no. 34 to the parliament with a value of €27 500 per QALY.^34^ To illustrate uncertainty surrounding the ICERs, bootstrapped (10 000 replicated datasets) cost and effect pairs were plotted on CE planes (CE planes) and CE acceptability curves (CEACs). A CE plane is divided into four quadrants: the northwest (NWQ), the northeast (NEQ), the southwest (SWQ) and the southeast (SEQ), representing all combinations of possible outcomes. If most incremental cost-effectiveness pairs (ICERs) are located in the NWQ, the intervention (SE or AE) is assumed to be more costly and less effective than the benchmark (UC). Whereas the NEQ indicates that the intervention is more costly and more effective than the benchmark, the SWQ that the intervention is less costly and less effective, and the SEQ that the intervention is less costly and more effective. The CEACs were used to demonstrate the probability that the intervention (SE or AE) is cost-effective in comparison to UC for a range of different WTP values.

#### Sensitivity analysis

To evaluate the credibility of the imputation procedure on missing values, two sensitivity analyses were performed on the main analysis: (1) complete-case analysis without adjustment for missing data and (2) without outliers. Outliers were identified with simple scatterplots by visual inspection and defined as patients with somewhat remarkably high total costs; 7 patients with costs ≥€20 772 at 0–3 months, and 13 patients with costs ≥€52 289 at 0–12 months. Of the outliers, 13 patients had costs related to primary care consultations, 11 to medical specialist consultations, 10 to sick leave, 6 to work assessment allowance, 4 to surgery and 3 to disability benefits. To evaluate the effect of not including transportation costs related to the interventions, a sensitivity analysis including an estimated average transportation cost was performed. Transportation costs per patient were estimated by multiplying the number of physiotherapy consultations in the intervention period by an estimated average transportation cost of €7 (based on a government-set rate of €0.35 per km for an estimated average round-trip distance of 20 km). To evaluate the appropriateness of analysing groups’ differences in costs (skewed data) with a parametric test (Student’s t-test), a sensitivity analysis was conducted with the Mann-Whitney U test. To evaluate the effect of single variables on the ICERs, a multiple one-way sensitivity analysis was performed. Relevant costs and QALYs were varied 20% below and above estimates used in the main analysis (total costs, 0–12 months), and Tornado diagrams were used to visually demonstrate the results.

#### Sample size

This study contains secondary analysis embedded in a three-arm RCT. Details on sample size calculation related the primary aim are provided elsewhere.^16 21^

### Patient and public involvement

This study was designed when user involvement was not a mandatory part of trials. Yet, the SE programme was developed in collaboration with clinicians with high competence in treating patients with knee OA. Results will be disseminated to the recruiting primary and secondary care providers and the participating patients.

## Results

A total of 168 patients were randomised into the three groups: 55 to SE, 56 to AE and 57 to UC. Seven participants (4%) withdrew their informed consent (due to lack of time or not attending tests), leaving 161 (96%) participants for the ITT analyses (54 to SE, 53 to AE and 54 to UC). Flow of participants through the study is shown in online supplemental additional file 2, figure A2. Table 2 shows patient characteristics and clinical status at baseline, along with the proportion of missing data per variable. Overall, the SE and AE groups were balanced as compared with the UC group, and there were no statistically significant differences in EQ-5D-5L scores between the SE or AE group as compared with the UC group. There were only minor differences between responders and non-responders (online supplemental additional file 3, table A1).

**Table 2:** Patient characteristics and clinical status at baseline

	All participants (n=161)	Missing, n (%)	Treatment groups		
			SE (n=54)	AE (n=53)	UC (n=54)
Male, n (%)	79 (49)	0 (0)	24 (44)	25 (47)	30 (56)
Age in years, mean (SD)	58 (7)	0 (0)	58 (7)	57 (7)	58 (7)
Body mass index, mean (SD)	29 (4)	1 (0.6)	29 (4)	29 (4)	28 (4)
Educational level high (>4 years), n (%)	42 (26)	0 (0)	15 (28)	15 (28)	12 (22)
Occupational status					
Working (paid work)	103 (64)	1 (0.6)	39 (73)	34 (64)	30 (55)
Sick leave/AAP	20 (13)	1 (0.6)	3 (6)	9 (17)	8 (15)
Disability pension	10 (6)	1 (0.6)	2 (4)	3 (6)	5 (9)
Age pension	24 (15)	1 (0.6)	8 (15)	7 (13)	9 (17)
Student/other	3 (2)	1 (0.6)	1 (2)	0 (0)	2 (4)
Pain severity average last week (NRS, 0–10), mean (SD)	5 (2)	1 (0.6)	5 (2)	5 (2)	5 (2)
Symptom duration in years, median (IQR)	5 (2–11)	0 (0)	4 (2–11)	5 (3–12)	4 (1–11)
Use of healthcare prior to inclusion*	59 (38)	4 (3)	17 (33)	19 (36)	23 (43)
Health related QOL (EQ-5D-5L, −0.59–1), mean (SD)	0.75 (0.19)	0 (0)	0.77 (0.16)	0.73 (0.19)	0.73 (0.20)

AE indicates group; EQ-5D-5L, EuroQols health-related quality of life measure; NRS, Numeric Rating Scale; , strength exercise group; UC, group. All percentage are presented by valid percentage of total. *last (general practitioner, physiotherapist, chiropractor, manual therapist, psychomotoric physiotherapist, other therapists, medical specialist)

* Last 3 months (general practitioner, physiotherapist, chiropractor, manual therapist, psychomotoric physiotherapist, other therapists and medical specialist).

AAPwork assessment allowanceAEaerobic exercise groupEQ-5D-5LEuroQol-5 Dimensions-5 LevelsNRSNumeric Rating ScaleSEstrength exercise groupUCusual care group

Missing data ranged from 0.0% to 3.0% for included baseline variables and 0.0% to 27.0% for follow-up variables (healthcare utilisation, productivity loss, EQ-5D-5L) used to calculate the outcome values.

### Healthcare utilisation, productivity loss, cost estimation and effect

Table 3 shows healthcare utilisation and productivity loss throughout 1 year of follow-up for all participants. Online supplemental additional file 4, tables A2–A4 show healthcare utilisation (including physiotherapist consultations related to the study interventions) and productivity loss throughout 1 year of follow-up for the three groups separately.

**Table 3:** Healthcare utilisation and productivity loss throughout 1 year of follow-up (n=161)

	0 to 3 months		>3 to 6 months		>6 to 9 months		>9 to 12 months	
	Missing, n (%)		Missing, n (%)		Missing, n (%)		Missing, n (%)	
Primary care								
Primary care consultation, n (%)		34 (21)		19 (12)		22 (14)		43 (27)
General practitioner	9 (7)		19 (13)		16 (12)		9 (8)	
Physiotherapist	25 (20)		38 (27)		37 (27)		28 (24)	
Chiropractor	1 (1)		0 (0)		0 (0)		1 (1)	
Manual therapist	3 (2)		0 (0)		1 (1)		6 (5)	
Psychomotoric physiotherapist	0 (0)		0 (0)		0 (0)		0 (0)	
Other therapists*	2 (2)		0 (0)		3 (2)		4 (3)	
No primary care consultations	93 (73)		96 (68)		93 (67)		77 (65)	
Numbers of consultations, median (IQR)†								
General practitioner	1 (1–1)	1 (11)	1 (1–2)	2 (11)	2 (1–2)	2 (13)	2 (1–3)	0 (0)
Physiotherapist	12 (5–24)	3 (12)	15 (6–24)	0 (0)	24 (12–24)	1 (3)	21 (12–24)	4 (14)
Chiropractor	6 (6–6)	0 (0)	–		–		3 (3–3)	0 (0)
Manual therapist	4 (1–)	0 (0)	–		2 (2–2)	0 (0)	4 (3–7)	0 (0)
Psychomotoric physiotherapist	–		–		–		–	
Other therapist*	2 (1–)	0 (0)	–		3 (2–)	0 (0)	8 (2–15)	0 (0)
Secondary care								
Medical specialist consultation, N (%)	5 (4)	34 (21)	10 (7)	19 (12)	13 (9)	22 (14)	4 (3)	43 (27)
Knee replacement surgery, n (%)	1 (1)	0 (0)	1 (1)	0 (0)	3 (2)	0 (0)	1 (1)	0 (0)
Productivity loss								
Sick leave, n (%)	15 (11)	19 (12)	9 (6)	15 (9)	13 (9)	17 (11)	11 (8)	28 (17)
Work assessment allowance, n (%)	4 (3)	19 (12)	8 (6)	15 (9)	9 (6)	17 (11)	8 (6)	28 (17)
Disability benefits, n (%)	4 (3)	19 (12)	4 (3)	15 (9)	4 (3)	17 (11)	4 (3)	28 (17)

Cells marked with a dash (-−) indicate that the variable was not reported.

* Naprapath, osteopath, acupuncture, masseuse.

† Numbers of consultations is calculated on basis of patients who have reported primary care consultations.

Mean (SD) intervention cost per patient was €190 (€433) and €201 (€580) for the SE and the AE intervention, respectively. Costs related to healthcare utilisation (including physiotherapist consultations related to the study interventions) and productivity loss for the 1 year of follow-up are shown in table 4. Online supplemental additional file 5, table A5 shows costs related to healthcare utilisation and productivity loss for the follow-up periods 0 to 3 months and >3 to 12 months. Costs were mainly related to productivity loss, accounting for 75%, 89% and 91% of total costs during 1 year of follow-up in the SE, AE and UC group, respectively. During the 1 year of follow-up, the mean cost per patient related to healthcare utilisation was approximately half in the SE group as compared with the AE and UC group (€848 vs €2003 and €1654). Whereas the mean cost per patient related to productivity loss was approximately half in the SE and AE group as compared with the UC group (€7105 and €6098 vs €15 745). In total, during the 1 year of follow-up, the sum of all costs was statistically significant lower in the SE and AE groups as compared with the UC group, with an estimated mean (95% CI) difference per patient at €−9445 (€−17 599 to €−1291) and €−9297 (€−17 223 to €−1371), respectively (table 5). The sensitivity analysis conducted with the Mann-Whitney U test provided similar results in terms of statistical significance.

**Table 4:** Costs (€) due to healthcare utilisation and productivity loss throughout 1 year of follow-up for all treatment groups*

Cost categories	SE	AE	UC
Primary care			
General practitioner	26 (44)	38 (56)	47 (60)
Physiotherapist	669 (1013)	1083 (1668)	1053 (1345)
Chiropractor	1 (3)	11 (67)	1 (4)
Manual therapist	55 (124)	62 (110)	64 (125)
Psychomotoric physiotherapist	0 (0)	0 (0)	0 (0)
Other therapists	66 (182)	66 (124)	91 (236)
Secondary care			
Medical specialist	31 (72)	45 (68)	56 (77)
Knee replacement surgery	0 (0)	698 (2466)	343 (1763)
Productivity loss (252 workdays pr year)			
Sick leave	4544 (138 001)	3460 (6646)	9339 (18 166)
Work assessment allowance	1461 (5803)	1517 (6142)	4756 (13 276)
Disability benefits	1100 (8086)	1121 (8162)	1650 (8972)
Total costs	7954 (17 659)	8101 (15 368)	17 398 (24 833)
Total costs healthcare utilisation	848 (1105)	2003 (3539)	1654 (2548)
Total cost productivity loss	7105 (17 371)	6098 (13 158)	15 745 (23 449)

AE indicates group; , strength exercise group; UC, group. Values are mean (SD) of costs (€).

* The presented estimates are pooled estimates based on the multiple imputation procedure.

AEaerobic exercise groupSEstrength exercise groupUCusual care group

**Table 5:** Mean cost (€) and effect (QALYs) differences (95% CI) between the intervention and usual care group during the period 0–3 and 0–12 months, including ICER, cost-effectiveness plane distributions and sensitivity analysis

	Δ costs (95% CI)	Δ QALYs (95% CI)	ICER	Distribution CE-plane (%)*			
				NEQ	SEQ	SWQ	NWQ
Strength exercise versus usual care							
Healthcare utilisation							
0–3 months†	24 (−116 to 165)	0.05 (−0.02 to 0.11)	480	54.5	33.7	3.2	8.6
0–12 months†	−805 (−1550 to −61)‡	0.05 (−0.02 to 0.12)	−16 100	1.5	88.8	9.5	0.2
Productivity loss							
0–3 months†	−1689 (−4081 to 704)	0.05 (−0.02 to 0.11)	−33 780	2.9	85.3	10.6	1.2
0–12 months†	−8639 (−16 453 to −825)‡	0.05 (−0.02 to 0.12)	−172 780	2.2	88.1	8.9	0.8
Total							
0–3 months†	−1664 (−4083 to 754)	0.05 (−0.02 to 0.11)	−33 280	3.0	85.2	10.5	1.3
0–12 months†	−9445 (−17 599 to −1291)‡	0.05 (−0.02 to 0.12)	−188 900	1.9	88.5	9.0	0.6
Sensitivity analysis (total costs)							
Complete case analysis							
0–3 months§	−1995 (−4739 to 748)	0.04 (−0.03 to 0.11)	−49 875				
0–12 months§	−11 842 (−20 858 to −2826)‡	0.05 (−0.02 to 0.13)	−236 840				
Without outliers							
0–3 months†¶	−1477 (−3108 to 154)	0.04 (−0.03 to 0.10)	−36 925				
0–12 months†¶	−3705 (−8232 to 821)	0.04 (−0.03 to 0.11)	−92 625				
Aerobic exercise versus usual care							
Healthcare utilisation							
0–3 months†	236 (−169 to 642)	0.02 (−0.06 to 0.09)	11 800	59.9	10.1	3.0	27.0
0–12 months†	349 (−834 to 1533)	0.04 (−0.04 to 0.11)	8725	54.5	32.5	3.1	9.9
Productivity loss							
0–3 months†	−2425 (−4581 to -269)‡	0.02 (−0.06 to 0.09)	−121 250	1.1	68.9	29.9	0.1
0–12 months†	−9647 (−16 969 to -2324)‡	0.04 (−0.04 to 0.11)	−241 175	1.5	85.6	12.6	0.3
Total							
0–3 months†	−2189 (−4475 to 97)	0.02 (−0.06 to 0.09)	−109 450	1.3	68.7	29.5	0.5
0–12 months†	−9297 (−17 223 to −1371)‡	0.04 (−0.04 to 0.11)	−232 425	1.9	85.2	12.3	0.6
Sensitivity analysis (total costs)							
Complete case analysis							
0–3 months§	−3393 (−5941 to −844)‡	0.02 (−0.06 to 0.09)	−169 650				
0–12 months§	−8894 (−20308 to 2521)	0.04 (−0.04 to 0.13)	−222 350				
Without outliers							
0–3 months†¶	−1512 (−3142 to 119)	0.01 (−0.06 to 0.08)	−151 200				
0–12 months†¶	−1938 (−6801 to 2925)	0.03 (−0.05 to 0.10)	−64 600				

CE-plane indicates cost-effectiveness plane; ICER, incremental cost-effectiveness ratio; NEQ, Northeast-Quadrant; NWQ, Northwest-Quadrant, SEQ, Southeast-Quadrant; SWQ, Southwest-Quadrant; QALY, quality-adjusted life year. QALYs are based on EuroQols health-related quality of life measure (EQ-5D-5L) with scores from −0.59 to 1. Higher scores indicatinge better quality of life. Differences are adjusted for baselines scores on sex, occupational status, healthcare utilizationutilisation prior to inclusion, and EQ-5D-5L. ICER = (cost intervention group - −cost usual care group) / (QALY intervention group - −QALY usual care group).

* Estimates based on bootstrapping (10 000 replicated datasets).

† Pooled estimates based on multiple imputation procedures.

‡ Statistic significant different between the two groups (p values≤0.03).

§ Complete -case analysis without adjustment for missing data.

¶ Sensitivity analysis excluding outliers (7 patients at 0–3 months, 13 patients at 0–12 months).

CE-planecost-effectiveness planeEQ-5D-5LEuroQol-5 Dimensions-5 LevelsICERincremental cost-effectiveness ratioNEQNortheast-QuadrantNWQNorthwest-QuadrantQALYquality-adjusted life yearSEQSoutheast-QuadrantSWQSouthwest-Quadrant

At all time points, there were no statistically significant difference in mean QALYs between the SE or AE group as compared with the UC group (table 5). The sensitivity analyses showed overall no substantial change in point estimates of mean cost (€) and effect (QALYs) differences when comparing complete case analysis, and analysis without outliers to the main analysis (table 5). Furthermore, no substantial change in point estimates of mean cost (€) and effect (QALYs) differences was seen when comparing analysis including transportation costs related to the intervention to the main analysis (online supplemental additional file 6, table A6).

### Cost-effectiveness

**Figure 1:**
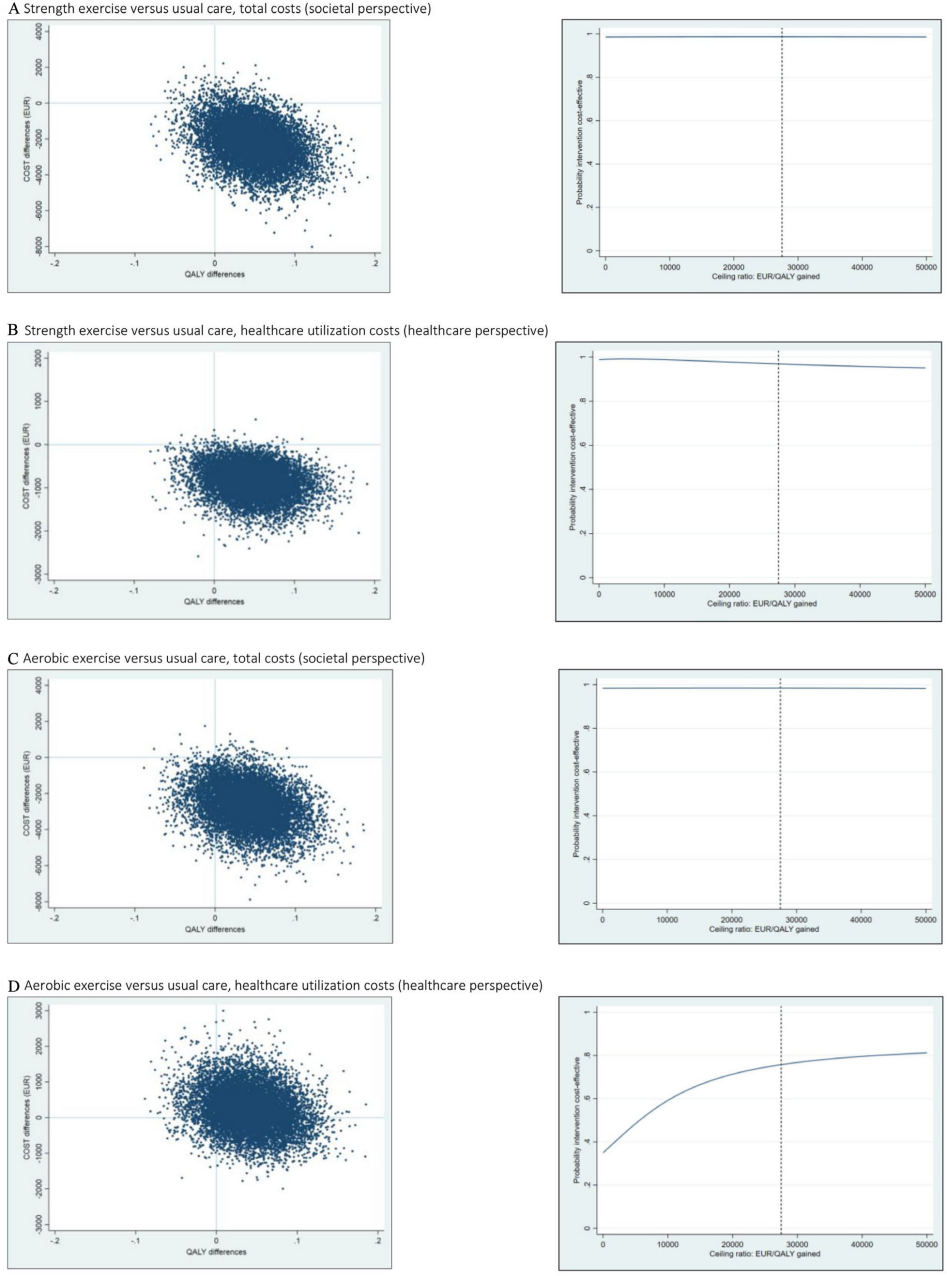
Cost-effectiveness plane and cost-effectiveness acceptability curve for different ceiling ratios (EUR) for quality-adjusted life-years (QALYs) indicating the probability of cost-effectiveness of strength exercise or aerobic exercise vs usual care on total costs (healthcare utilisation and productivity loss) and healthcare utilisation costs at 0–12 months. All estimates are based on bootstrapping (10 000 replicated datasets). The dashed line represents the willingness to pay (WTP) threshold of €27 500. EUR, euros.

Comparing SE to UC at 1 year of follow-up, all estimated ICERs were found to be with negative values, thus indicating that SE on average was less costly and more effective for improving QALYs than UC, both from a societal and a healthcare perspective. The CE planes showed similar results with most incremental CE pairs (86%–89%) being located in the SEQ (figure 1 and table 5). The CEACs indicated that the probability of SE being cost-effective compared with UC was ≥95% for all WTP thresholds.

Comparing AE to UC at 1 year of follow-up, we found a greater variation in the results. From a societal perspective, the estimated ICERs were found to be with a negative value, thus indicating that AE on average was less costly and more effective for improving QALYs than UC. The CE plane showed likewise that most incremental CE pairs (85%) were located in the SEQ (figure 1 and table 5). Furthermore, the CEAC indicated that the probability of AE being cost-effective compared with UC was ≥98% for all WTP thresholds. From a healthcare perspective, however, the estimated ICER was €8725 per QALY gained. The CE plane showed that most incremental CE pairs (55%) were located in the NEQ (figure 1 and table 5), thus indicating that AE on average was most likely to be more costly but also more effective for improving QALYs than UC. The CEAC indicated that the probability of AE being cost-effective compared with UC was ≥35 to 81% depending on the WTP threshold. Online supplemental additional file 7, figure A3 shows CE planes and CEACs for 3 months follow-up.

**Figure 2:**
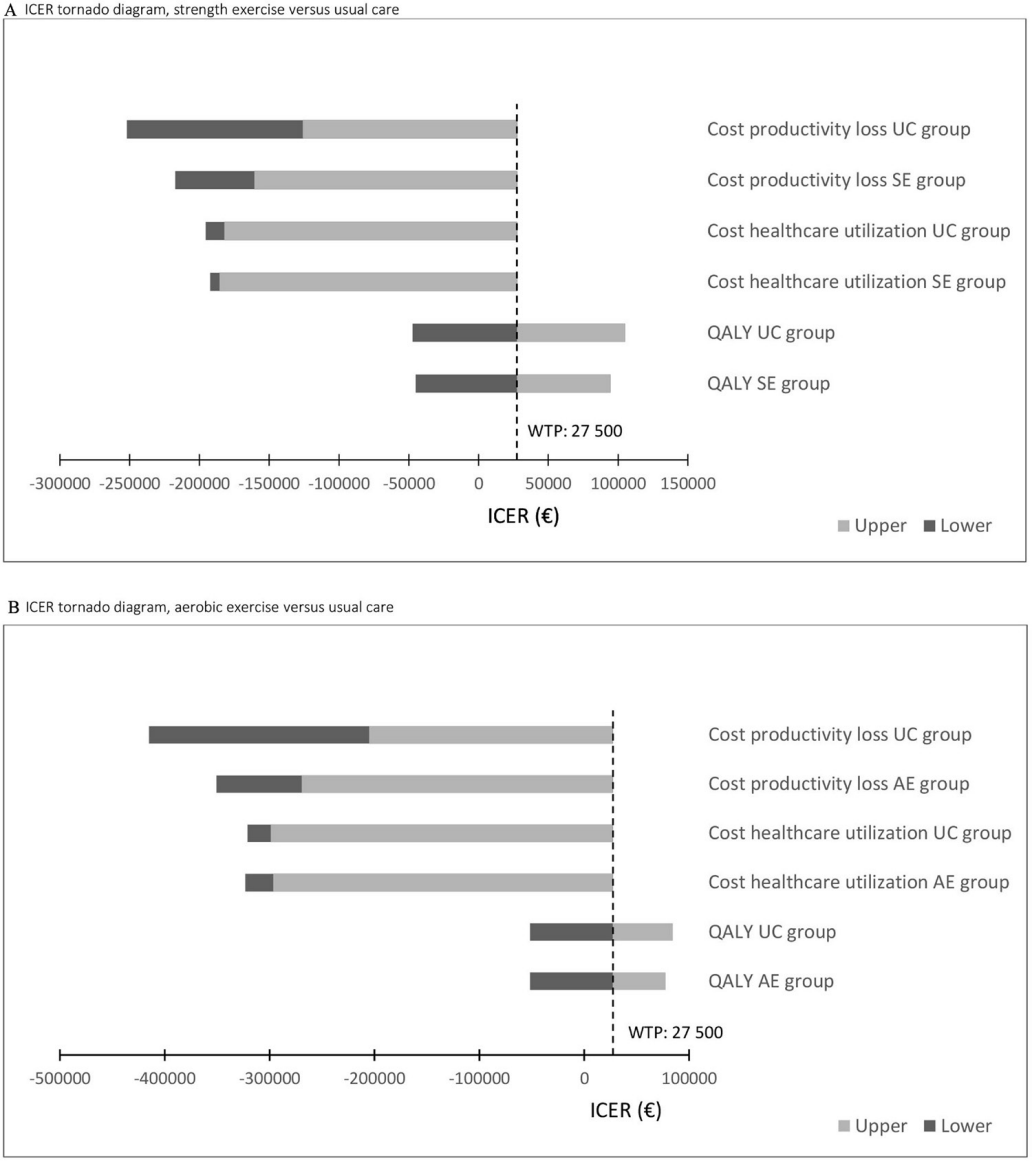
Incremental cost-effectiveness ratio (ICER) tornado diagram for multiple one-way sensitivity analyses of (A) strength exercise (SE) versus usual care (UC) and (B) aerobic exercise (AE) versus usual care. The tail of each bar indicates the upper (light grey) and lower (dark grey) bound of the ICER when relevant costs/QALYs is varied 20% below and above estimates used in the main analysis (total costs, 0–12 months). The dashed line represents the willingness-to-pay (WTP) threshold to provide a reference for the ICERs. QALYs, quality-adjusted life-years.

The multiple one-way sensitivity analyses illustrated that altering costs 20% below and above estimates used in the main analysis did not affect the ICERs significantly (figure 2). SE and AE were still considered to be cost-effective compared with UC (total costs, 0–12 months). Altering QALYs, however, had a significant effect on the ICERs and led to some ICERs above a WTP threshold of €27 500 per QALY gained.

## Discussion

The present study evaluated the 1-year CE of 12 weeks of SE or AE compared with UC for patients with symptomatic knee OA. From a societal and healthcare perspective, both SE and AE were found to be cost-effective at a WTP threshold of €27 500. Three out of four calculations at 1 year resulted in negative ICERs, indicating cost-saving interventions. Whereas AE from a healthcare perspective resulted in an ICER of €8725 per QALY gained. Although the results were sensitive to different estimates of QALYs (due to very small group differences circling around zero), all ICERs were well below the WTP threshold of €27 500. From a societal perspective, the probability of SE or AE being cost-effective was ≥98% for all WTP thresholds. From a healthcare perspective, the probability of SE being cost-effective was ≥95% for all WTP thresholds, whereas the probability of AE being cost-effective varied between 35% and 81%, depending on the WTP threshold.

Direct comparability of this study with other studies is limited due to no similar study has been conducted within the Norwegian healthcare system. Furthermore, there is a great heterogeneity in the method used among CE studies on knee OA.^15^ Nevertheless, our findings are generally in accordance with previous research from other countries. In a recent systematic review,^15^ overall, exercise interventions with or without education and diet as adjunctive therapies for hip and/or knee OA were found to be cost-effective at conventional WTP thresholds in numerous healthcare systems. Yet, CE seems to depend on (among other things) the comparator. 15 out of 16 studies included in the systematic review indicated that exercise interventions (4 with education and 2 with diet) were cost saving or cost-effective compared with education or physician-delivered UC, whereas three studies indicated that exercise interventions were not cost-effective compared with physiotherapist-delivered UC.^15^ Of studies reporting cost-saving or CE, ICERs ranged from cost-saving to US$883 per QALY gained (2019 US dollar).^15^ Moreover, three studies from the Nordic countries^35^,^37^ published after the systematic review of Mazzei *et al*^15^ also reported that exercise interventions with or without education and diet as adjunctive therapies for hip and/or knee OA is cost-effective.

With estimated annular costs related to lower-limb OA as high as €817 billion in Europe alone^5^ and an expected increase in the future burden of the disease,^1 2^ cost-effective treatment strategies is highly needed.^6 11 15^ Our study adds to the body of evidence that implementation of structured exercise programmes in clinical practice is a cost-effective alternative to UC in a 1-year horizon.

The main limitation of the present study is that it was powered on the primary outcome of the three-arm RCT, not on the CE outcome. However, this is common practice in trial-based economic evaluations, due to the right-skewed distribution of cost data and the fact that an extremely large sample size is required to detect relevant cost differences, which in turn can be both infeasible and/or unethical.^13^ To address this limitation, as recommended, bootstrapped estimates were used for result interpretation.^13^ A second limitation of the present study is that we had missing data on variables (healthcare utilisation, productivity loss, EQ-5D-5L) used to estimate the outcome variables and had to replace missing values. This might have introduced some risk of bias. Yet, there were only minor differences between responders and non-responders at baseline, an appropriate method of imputation (multiple imputation)^38^ was used, and sensitivity analyses showed similar results when comparing complete-case analysis to the main analysis. A third limitation is the fact that we expect to have somewhat underestimated total healthcare utilisation and related costs. Self-reports tend to underestimate the true value of healthcare utilisation due to potential recall bias.^39^,^42^ Moreover, we lack data on medication use and diagnostic imaging. Nevertheless, we consider the impact of this to be of only minor importance in this study, as healthcare utilisation and related costs were measured equally in the three groups being compared. Furthermore, the majority of costs in this study were related to productivity loss; costs due to healthcare utilisation had only a minor impact. A fourth potential limitation is that productivity loss was self-reported and not measured with a standardised validated method. However, a recent meta-analysis supports satisfactory agreement between self-reported and registry data on the occurrence and duration of absenteeism.^43^ Also, we lack data on productivity loss related to reduced productivity while at paid work (presenteeism) and unpaid work, thus also expect to have somewhat underestimate the true value of productivity loss, especially among people not in paid work. Yet, we also consider the impact of this to be of only minor importance in this study, as productivity loss and related costs were measured equally in the three groups being compared. A fifth limitation is the lack of data on eligible participants that declined to participate or for other reasons were not invited. Due to limited resources and practical reasons related to recruitment from a broad network of clinicians, it was not possible to record information on all eligible participants during the data collection period. Thus, the risk of selection bias is present.

The main strength of the present study is that it was a CE study of a three-arm RCT evaluating the effect of two structured exercise programmes with relatively few exercises involved and high adherence,^16^ that it was conducted in line with the Panel on Cost-Effectiveness in Health and Medicine guidelines,^19 20^ preplanned with a published statistical analysis plan and reported in line with the CHEERS Statement.^23^ Also, bootstrapping methods were used to determine uncertainty around the ICERs, which is recommended to handle skewed cost data.^44^

### Conclusion

In conclusion, the present study demonstrated that implementing SE or AE for patients with symptomatic knee OA is a cost-effective alternative compared with UC within the Norwegian healthcare system, at a WTP threshold of €27 500. Both are from a societal and healthcare perspective. Yet, the results were sensitive to different estimates of QALYs (due to very small group differences). Moreover, the result with regard to AE evaluated from a healthcare perspective should be interpreted with caution, due to some uncertainty surrounding the calculated ICER. Further studies are needed to confirm these findings. However, the present study gives further support to the notion that structured exercise programmes for patients with symptomatic knee OA have the potential to reduce the global burden of knee OA.

## supplementary material

10.1136/bmjopen-2023-079704 online supplemental file 1

## Data Availability

Data are available on reasonable request.
