# Supplementary material for: Cost-effectiveness of strength exercise or aerobic exercise compared with usual care for patients with knee osteoarthritis: secondary results from a multiarm randomised controlled trial in Norway
Source: BMJ Open. 2024 May 23;14(5):e079704. doi: 10.1136/bmjopen-2023-079704 (PMC11328638; doi:10.1136/bmjopen-2023-079704)
Supplement: online supplemental file 1 [file bmjopen-14-5-s001.pdf]

Additional file 1:

Questions Related to Employment Status and Healthcare Utilization
(Freely translate from Norwegian to English)

Employment Status

What is your current employment status?

☐ Income-generating employment ______ % (percentage of full-time employment)
☐ Student
☐ Engaged in unpaid work (e.g., household duties)
☐ Unemployed
☐ Retired pensioner
☐ Sick leave ______ % sick leave ______ (date)
  Was your sick leave due to knee osteoarthritis, please indicate: Yes / No
☐ Receiving work assessment allowance
  Reason for receiving work assessment allowance: ____________________________________
☐ Disability pensioner ______ %
  If disability pensioner due to the knee condition, from when (date): ______
☐ Other, please specify: _____________________________________

If you were on sick leave when you responded three months ago, when did you return to full-time work? Date: ______

If you did not return to full-time work, what percentage were you on sick leave and when did you return to part-time work? Date: ______ Sick leave: ______ %

Healthcare utilization

In the past 3 months, have you sought any treatment for your knee issues? If Yes, please indicate the type of treatment and the number of consultations

☐ No, none	
☐ General practitioner	Number of consultations: _______
☐ Medical specialist	Number of consultations: _______
☐ Chiropractor	Number of consultations: _______
☐ Physiotherapist	Number of consultations: _______
☐ Manual therapist	Number of consultations: _______
☐ Psychomotoric physiotherapist	Number of consultations: _______
☐ Acupuncture	Number of consultations: _______
☐ Other, specify: _______	Number of consultations: _______

Figure A1. Questions used to measure Employment Status and Healthcare Utilization

Additional file 2:

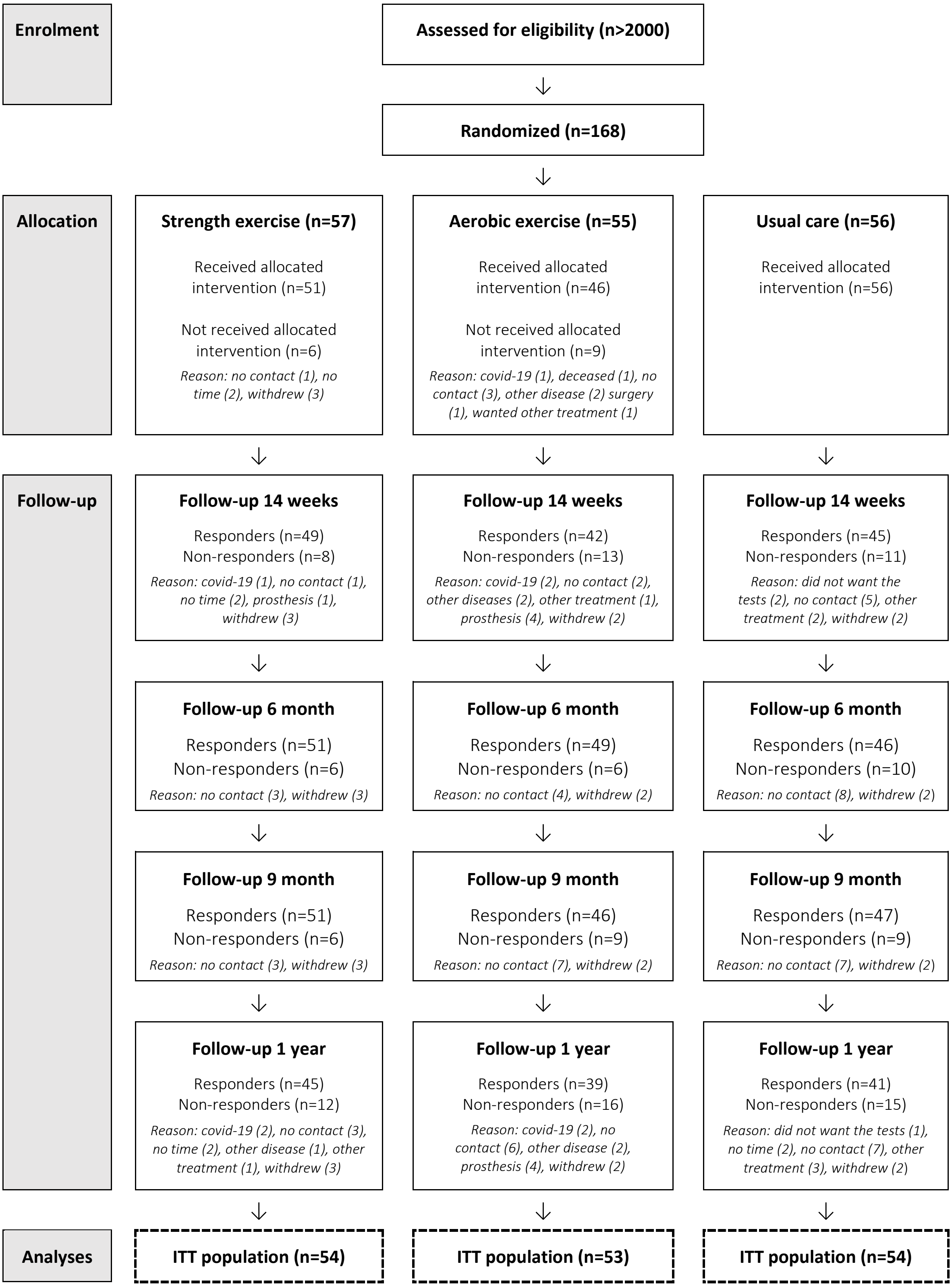

Figure A2. Participant flow chart. ITT indicates intention to treat

Additional file 3:

Table A1. Patient characteristics and clinical status at baseline, responders (all timepoints) versus non-responders (at minimum 1 timepoint)

	**Responders (n = 100)**	**Non-responders (n = 61)**
Male, n (%)	50 (50)	29 (48)
Age in years, mean (SD)	58 (7)	57 (7)
Body mass index, mean (SD)	29 (4)	29 (4)
Educational level high (> 4 years), n (%)	27 (27)	15 (25)
Occupational status		
Working (paid work)	63 (63)	40 (67)
Sick leave / AAP	11 (11)	9 (15)
Disability pension	6 (6)	4 (7)
Age pension	18 (18)	6 (10)
Student/Other	2 (2)	1 (2)
Pain severity average last week (NRS, 0-10), mean (SD)	5 (2)	5 (2)
Symptom duration in years, median (IQR)	5 (2-11)	5 (1-12)
Use of healthcare prior to inclusion*	36 (37)	23 (39)
Health related QOL (EQ-5D-5L, -0.59-1), mean (SD)	0.75 (0.17)	0.74 (0.20)

NRS indicates Numeric Rating Scale; EQ-5D-5L, EuroQol's health-related quality of life measure. All percentage are presented by valid percentage of total. *last 3 months (general practitioner, physiotherapist, chiropractor, manual therapist, psychomotoric physiotherapist, other therapists, medical specialist)

Additional file 4:

Table A2. Healthcare utilization and productivity loss throughout one-year of follow-up, strength exercise group (n = 54)

	0-3 months		>3-6 months		>6-9 months		>9-12 months	
		**Missing, n (%)**		**Missing, n (%)**		**Missing, n (%)**		**Missing, n (%)**
Primary care								
Primary care consultation, n (%)		5 (9)		3 (6)		3 (6)		12 (22)
General practitioner	3 (6)		4 (8)		7 (14)		3 (7)	
Physiotherapist	11 (22)		11 (22)		9 (18)		5 (12)	
Chiropractor	0 (0)		0 (0)		0 (0)		0 (0)	
Manual therapist	1 (2)		0 (0)		1 (2)		4 (10)	
Psychomotoric physiotherapist	0 (0)		0 (0)		0 (0)		0 (0)	
Other therapists**	1 (2)		0 (0)		1 (2)		2 (5)	
No primary care consultations	36 (74)		39 (77)		36 (71)		31 (74)	
Numbers of consultations, median (IQR)*								
General practitioner	1 (1-1)	1 (33)	1 (1-2)	0 (0)	1 (1-2)	2 (29)	1 (1-)	0 (0)
Physiotherapist	18 (5-32)	1 (9)	12 (2-24)	0 (0)	18 (12-24)	0 (0)	18 (11-24)	0 (0)
Chiropractor	-		-		-		-	
Manual therapist	1 (1-1)	0 (0)	-		2 (2-2)	0 (0)	4 (2-9)	0 (0)
Psychomotoric physiotherapist	-		-		-		-	
Other therapist**	3 (3-3)	0 (0)	-		3 (3-3)	0 (0)	9 (1-)	0 (0)
Secondary care								
Medical specialist consultation, N (%)	1 (2)	5 (9)	3 (6)	3 (6)	2 (4)	3 (6)	3 (7)	12 (22)
Knee replacement surgery, n (%)	0	0 (0)	0	0 (0)	0	0 (0)	0	0 (0)
Productivity loss								
Sick leave, n (%)	5 (10)	4 (7)	3 (6)	3 (6)	3 (6)	3 (6)	2 (4)	8 (15)
Work assessment allowance, n (%)	1 (2)	4 (7)	2 (4)	3 (6)	2 (4)	3 (6)	2 (4)	8 (15)
Disability benefits, n (%)	1 (2)	4 (7)	1 (2)	3 (6)	1 (2)	3 (6)	1 (2)	8 (15)

Cells marked with a dash (-) indicate that the variable was not reported. *Numbers of consultations is calculated on basis of patients who have reported primary care consultations. **Naprapath, osteopath, acupuncture, masseuse.

Table A3. Healthcare utilization and productivity loss throughout one-year of follow-up, aerobic exercise group (n = 53)

	0-3 months		>3-6 months		>6-9 months		>9-12 months	
		Missing, n (%)		Missing, n (%)		Missing, n (%)		Missing, n (%)
Primary care								
Primary care consultation, n (%)		14 (26)		7 (13)		10 (19)		15 (28)
General practitioner	3 (8)		6 (13)		4 (9)		2 (5)	
Physiotherapist	4 (10)		15 (33)		11 (26)		9 (24)	
Chiropractor	1 (3)		0 (0)		0 (0)		1 (3)	
Manual therapist	0 (0)		0 (0)		0 (0)		2 (5)	
Psychomotoric physiotherapist	0 (0)		0 (0)		0 (0)		0 (0)	
Other therapists**	0 (0)		0 (0)		1 (2)		1 (3)	
No primary care consultations	32 (82)		30 (65)		30 (70)		24 (63)	
Numbers of consultations, median (IQR)*								
General practitioner	1 (1-1)	0 (0)	1 (1-2)	0 (0)	2 (1-3)	0 (0)	2 (2-2)	0 (0)
Physiotherapist	24 (6-)	1 (25)	18 (8-24)	0 (0)	24 (6-36)	0 (0)	24 (15-24)	2 (22)
Chiropractor	6 (6-6)	0 (0)	-		-		3 (3-3)	0 (0)
Manual therapist	-		-		-		5 (3-)	0 (0)
Psychomotoric physiotherapist	-		-		-		-	
Other therapist**	-		-		4 (4-4)	0 (0)	4 (4-4)	0 (0)
Secondary care								
Medical specialist consultation, N (%)	2 (5)	14 (26)	3 (7)	7 (13)	6 (14)	10 (19)	0 (0)	15 (28)
Knee replacement surgery, n (%)	1 (2)	0 (0)	1 (2)	0 (0)	2 (4)	0 (0)	0 (0)	0 (0)
Productivity loss								
Sick leave, n (%)	2 (4)	8 (15)	1 (2)	4 (8)	4 (9)	7 (13)	4 (9)	10 (19)
Work assessment allowance, n (%)	0 (0)	8 (15)	2 (4)	4 (8)	2 (4)	7 (13)	1 (2)	10 (19)
Disability benefits, n (%)	1 (2)	8 (15)	1 (2)	4 (8)	1 (2)	7 (13)	1 (2)	10 (19)

Cells marked with a dash (-) indicate that the variable was not reported. *Numbers of consultations is calculated on basis of patients who have reported primary care consultations. **Naprapath, osteopath, acupuncture, masseuse.

able A4. Healthcare utilization and productivity loss throughout one-year of follow-up, usual care group (n = 54)

	0-3 months		>3-6 months		>6-9 months		>9-12 months	
		Missing, n (%)		Missing, n (%)		Missing, n (%)		Missing, n (%)
Primary care								
Primary care consultation, n (%)		15 (28)		9 (17)		9 (17)		16 (29)
General practitioner	3 (8)		9 (20)		5 (11)		4 (11)	
Physiotherapist	10 (26)		12 (27)		17 (38)		14 (37)	
Chiropractor	0 (0)		0 (0)		0 (0)		0 (0)	
Manual therapist	2 (5)		0 (0)		0 (0)		0 (0)	
Psychomotoric physiotherapist	0 (0)		0 (0)		0 (0)		0 (0)	
Other therapists**	1 (3)		0 (0)		1 (2)		1 (3)	
No primary care consultations	25 (36)		27 (60)		27 (60)		22 (58)	
Numbers of consultations, median (IQR)*								
General practitioner	1 (1-1)	0 (0)	1 (1-2)	2 (22)	2 (1-3)	0 (0)	2 (1-3)	0 (0)
Physiotherapist	8 (2-12)	1 (10)	15 (7-24)	0 (0)	24 (12-24)	1 (6)	14 (7-24)	2 (14)
Chiropractor	-		-		-		-	
Manual therapist	6 (4-)	0 (0)	-		-		-	
Psychomotoric physiotherapist	-		-		-		-	
Other therapist**	1 (1-1)	0 (0)	-		2 (2-2)	0 (0)	12 (12-12)	0 (0)
Secondary care								
Medical specialist consultation, N (%)	2 (5)	15 (28)	4 (9)	9 (17)	5 (11)	9 (17)	1 (3)	16 (29)
Knee replacement surgery, n (%)	0 (0)	0 (0)	0 (0)	0 (0)	1 (2)	0 (0)	1 (2)	0 (0)
Productivity loss								
Sick leave, n (%)	8 (17)	7 (13)	5 (11)	8 (15)	6 (13)	7 (13)	5 (11)	10 (19)
Work assessment allowance, n (%)	3 (6)	7 (13)	4 (9)	8 (15)	5 (11)	7 (13)	5 (11)	10 (19)
Disability benefits, n (%)	2 (4)	7 (13)	2 (4)	8 (15)	2 (4)	7 (13)	2 (4)	10 (19)

Cells marked with a dash (-) indicate that the variable was not reported. *Numbers of consultations is calculated on basis of patients who have reported primary care consultations. **Naprapath, osteopath, acupuncture, masseuse.

Additional file 5:

Table A5. Costs (€) due to healthcare utilization and productivity loss from 0-3 months and >3-12 months for all treatment groups*

	0-3 months			>3-12 months		
Cost categories	SE	AE	UC	SE	AE	UC
Primary care						
General practitioner	3 (9)	5 (12)	6 (12)	24 (42)	33 (51)	41 (57)
Physiotherapist	190 (433)	201 (580)	126 (239)	479 (876)	883 (1220)	927 (1235)
Chiropractor	1 (2)	7 (45)	1 (2)	0.3 (1)	4 (23)	0.4 (1)
Manual therapist	6 (19)	17 (40)	31 (94)	49 (124)	45 (89)	32 (64)
Psychomotoric physiotherapist	0 (0)	0 (0)	0 (0)	0 (0)	0 (0)	0 (0)
Other therapists	6 (31)	6 (12)	8 (16)	60 (180)	60 (120)	84 (231)
Secondary care						
Medical specialist	4 (15)	10 (22)	13 (31)	28 (62)	35 (55)	41 (70)
Knee replacement surgery	0 (0)	175 (1270)	0 (0)	0 (0)	524 (2158)	343 (1763)
Productivity loss (252 workdays pr year)						
Sick leave	1714 (4937)	825 (2265)	2582 (5809)	2830 (9999)	2635 (6815)	6756 (14 806)
Work assessment allowance	255 (1129)	402 (1506)	937 (3008)	1207 (5086)	1115 (4717)	3818 (10 778)
Disability benefits	275 (2022)	280 (2041)	413 (2243)	825 (6065)	841 (6121)	1238 (6729)
**Total costs**	**2452 (5483)**	**1928 (4508)**	**4117 (6684)**	**5501 (12 691)**	**6173 (11 781)**	**13 282 (19 174)**
Total costs healthcare utilization	209 (434)	421 (1468)	185 (225)	639 (985)	1582 (2834)	1469 (2483)
Total cost productivity loss	2243 (5393)	1507 (3675)	3932 (6616)	4862 (12 480)	4591 (10 264)	11 813 (18 288)

AE indicates aerobic exercise group; SE, strength exercise group; UC, usual care group. Values are mean (SD) of costs (€). *The presented estimates are pooled estimates based on the multiple imputation procedure.

Additional file 6:

Table A6. Sensitivity analysis including transportation costs related to the interventions. Mean cost (€) and effect (QALYs) differences (95% CI) between the intervention and usual care group during the period 0-3 and 0-12 months, including ICER

	**Δ costs (95% CI)**	**Δ QALYs (95% CI)**	**ICER**
**Strength exercise versus usual care**			
Healthcare utilization			
0-3 months[a,b]	71 (-113 to 255)	0.05 (-0.02 to 0.11)	1440
0-12 months[a,b]	-877 (-1893 to 193)	0.05 (-0.02 to 0.12)	-17 540
Total			
0-3 months[a,b]	-1983 (-4836 to 870)	0.05 (-0.02 to 0.11)	-39 660
0-12 months[a,b]	-10 279 (-20 234 to -324)*	0.05 (-0.02 to 0.12)	-205 580
**Aerobic exercise versus usual care**			
Healthcare utilization			
0-3 months[a,b]	27 (-226 to 279)	0.02 (-0.06 to 0.09)	1350
0-12 months[a,b]	47 (-1590 to 1522)	0.04 (-0.04 to 0.11)	1175
Total			
0-3 months[a,b]	-3387 (-5940 to -833)*	0.02 (-0.06 to 0.09)	-169 350
0-12 months[a,b]	-11 563 (-21 254 to -2051)*	0.04 (-0.04 to 0.11)	-289 075

ICER indicates incremental cost-effectiveness ratio; QALY, quality-adjusted life year. QALYs are based on EuroQol's health-related quality of life measure (EQ-5D-5L) with scores from −0.59 to 1. Higher scores indicating better quality of life. Differences are adjusted for baselines scores on sex, occupational status, healthcare utilization prior to inclusion, and EQ-5D-5L. ICER = (cost intervention group - cost usual care group) / (QALY intervention group - QALY usual care group). [a]Pooled estimates based on multiple imputation procedures. [b]Sensitivity analysis including transportation costs related to the interventions (estimated average, €7 per physiotherapy consultation in the intervention period). *Statistic significant different between the two groups (p values ≤ 0.03).

Additional file 7:

(a) Strength exercise versus usual care, total costs 0-3 months (societal perspective)

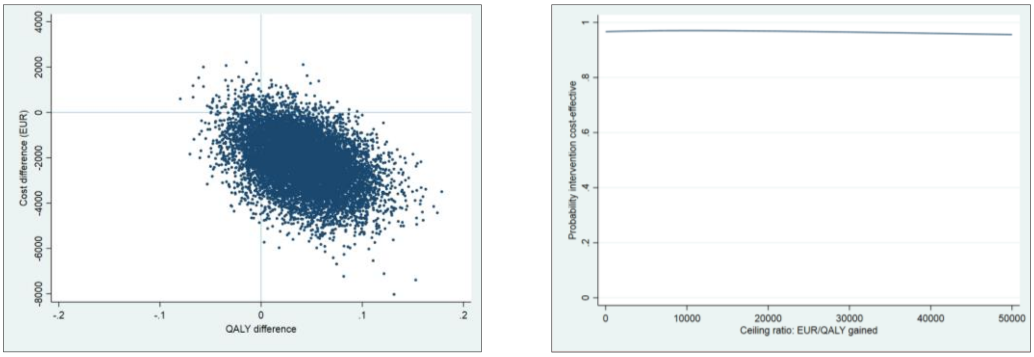

(b) Strength exercise versus usual care, healthcare utilization costs 0-3 months (healthcare perspective)

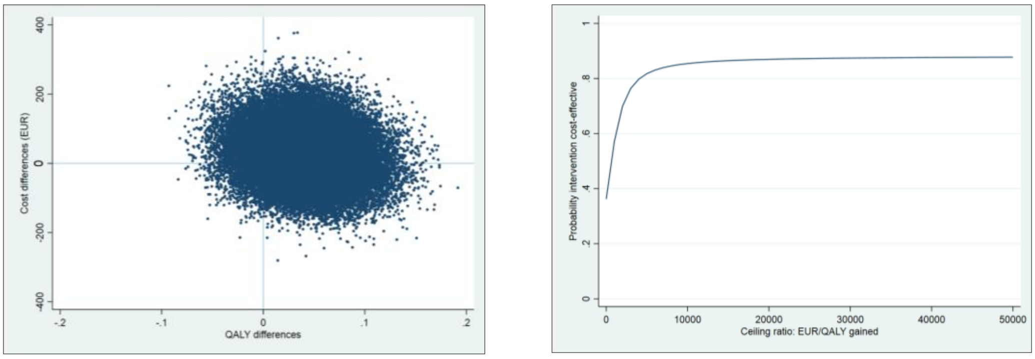

(c) Aerobic exercise versus usual care, total costs 0-3 months (societal perspective)

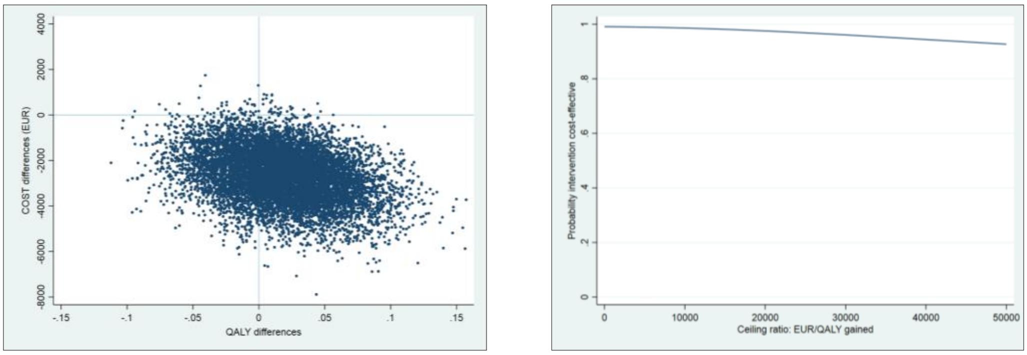

(d) Aerobic exercise versus usual care, healthcare utilization costs 0-3 months (healthcare perspective)

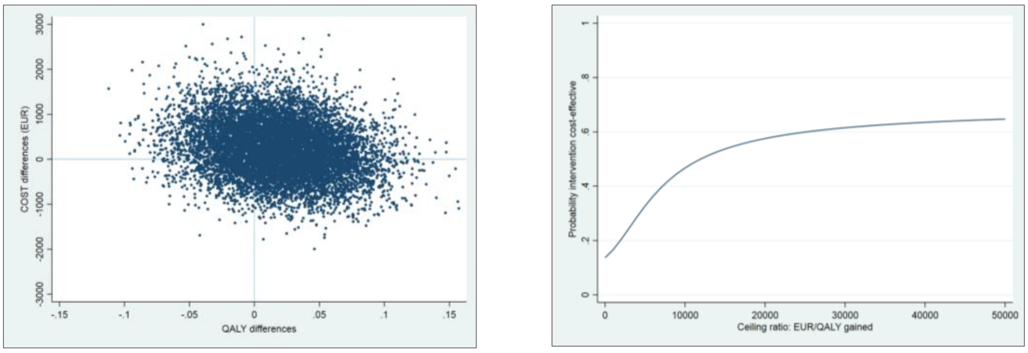

Figure A3. Cost-effectiveness plane and cost-effectiveness acceptability curve for different ceiling ratios (EUR) for quality adjusted life-years indicating the probability of cost-effectiveness of strength exercise or aerobic exercise versus usual care on total costs (healthcare utilization and productivity loss) and healthcare utilization costs at 0-3 months. All estimates are based on bootstrapping (10 000 replicated datasets). EUR indicates euros; QALY, quality-adjusted life years.
