# Supplementary material for: Cost-effectiveness of strength exercise or aerobic exercise compared with usual care for patients with knee osteoarthritis: secondary results from a multiarm randomised controlled trial in Norway
Source: BMJ Open. 2024 May 23;14(5):e079704. doi: 10.1136/bmjopen-2023-079704 (PMC11328638; doi:10.1136/bmjopen-2023-079704)
Supplement: Supplementary file 2 [file bmjopen-2023-079704.reviewer_comments.pdf]

PEER REVIEW HISTORY

BMJ Open publishes all reviews undertaken for accepted manuscripts. Reviewers are asked to complete a checklist review form (**http://bmjopen.bmj.com/site/about/resources/checklist.pdf**) and are provided with free text boxes to elaborate on their assessment. These free text comments are reproduced below.

ARTICLE DETAILS

**TITLE (PROVISIONAL)**	Cost-effectiveness of strength exercise or aerobic exercise compared to usual care for patients with knee osteoarthritis: secondary results from a multi-arm randomised controlled trial in Norway
**AUTHORS**	Killingmo, Rikke (proxy) (contact); Øiestad, Britt; Risberg, May-Arna; Maas, Esther; Grotle, Margreth

VERSION 1 – REVIEW

**REVIEWER**	Okafor, Charles<br>The University of Queensland, Centre for Health Services Research
**REVIEW RETURNED**	22-Jan-2024

**GENERAL COMMENTS**	I thank the authors for this work, and I find the paper interesting. My major concerns is around the estimation of cost. The cost components in Table 1 does not seem to capture all relevant cost. It is good for readers to have a comprehensive view of the components for replicability of this works elsewhere.<br>- What about the cost exercise at home? Purchase of exercise equipment?<br>- Cost of registration/attendance of gymnasium for home exercise.<br>- What major cost components constitutes the “usual care”? Readers need to know because it is unclear at the moment. Medications taken e.g., NSAIDS taken? And Duration?<br>- Given that the QALY are similar, and ‘cost’ is the major driver of the cost saving estimated in the study. The authors need to be clear and spot-on on the cost components.<br>- Why should transportation costs be taken off? This cost is required to measure the societal cost of the intervention.<br>- The authors need to be explicit on what constitutes the cost components of each intervention.<br>- It will be informative to see the healthcare utilisation for each intervention/comparator to know how the different groups utilised healthcare.

**REVIEWER**	Pinheiro , Marina<br>University of Sydney
**REVIEW RETURNED**	12-Feb-2024

**GENERAL COMMENTS**	Thank you very much for the opportunity to review this manuscript.<br><br>Cost-effectiveness investigations are relatively rare in this field, so this manuscript brings an important contribution to the field. Overall, the economic evaluation was well-performed and reported however in several aspects it lacks details. Additionally, the

	nuances in the interpretation of results need to be considered and clearly stated. I have added suggestions below that aim to improve the clarity of the manuscript.<br><br>Abstract:<br>1) I would suggest that you include the ICERs with Bootstrapped confidence intervals.<br>2) It would be helpful to state that the main timepoint for the analysis was 12 months.<br><br>Methods:<br>3) The authors described that healthcare utilization and productivity loss data were collected at 6 and 9 months. If the time horizon for this study was 12 months, how was healthcare utilisation collected for the last 3 months of the trial (month 10 to 12)?<br><br>4) Which instrument was used to collect healthcare utilization and productivity loss? If a validated questionnaire was not used (which seems to be the case), I would suggest that the authors include the instruments as supplement materials.<br><br>5) Why did the authors restrict healthcare utilisation to utilization due to knee OA? One limitation of this approach is that the intervention might have benefits or harms that expand beyond what would have been captured by "healthcare utilisation due to knee". It would be helpful to add this point to the discussion.<br><br>6) Was the person conducting the interviews blinded to group allocation? The authors state that group allocation was concealed for the assessors, but it would be helpful to make it clearer if this also applies to the people conducting the interviews to collect information on healthcare utilisation and productivity.<br><br>7) The recall bias is an important limitation of this study and should be discussed.<br><br>8) What was the location for the exercise sessions? Were participants in the AE group given a stationary bike to take home? Or did they have to travel to a gym? Were physiotherapists the only professionals supervising the exercise classes? Did participants exercise in groups? Or individually? Although the authors cited a reference where the details of the intervention have been described, it would be helpful to have this information described as they have an impact on costs.<br><br>9) How were intervention costs calculated? It seems that only physiotherapy staff costs were captured as part of the resource utilization data collection. Were there other costs involved with implementing the intervention in terms of equipment/materials, travel time, rental (location)? These need to be considered and reported.<br>10) I would suggest that authors discuss the limitations of valuing productivity according to employment rate, particularly considering people who might not be at paid work.<br><br>11) Was the identification of outliers for the sensitivity analysis conducted blinded to group? As a specific criterion was not set for defining an outlier it is possible that group allocation knowledge could impact on the judgement.

	12) It is usually recommended that conversion of currencies be conducted using purchasing power parity conversion factors.<br><br>Results<br>13) It would be helpful if bootstrapped 95% CI intervals could be reported for the ICER measures.<br><br>14) Intervention costs should be reported separately. A detailed breakdown of the intervention costs would be very helpful to inform people who are planning to implement a similar program in the future.<br><br>15) Figure 1: it would be helpful to indicate the selected WTP threshold in the acceptability curves in Figure 1 (perhaps with a dotted line?).<br><br>16) Fig 1A. The acceptability curve indicates that the intervention has close to a 100% probability of being cost-effective considering various WTP thresholds. However, looking at the cost-effectiveness plane there are quite a few points where the intervention was more costly (north quadrant) and more (NE) or less effective (NW) than usual care. These results don't seem to match what is presented in the acceptability curve. I would only expect a flat line in a situation where all points are located in the southeast quadrant. Similarly, Fig 1B shows that the probably of an intervention being cost-effective decreases as WTP increases which is contra-intuitive. I would suggest that authors check these results.<br><br>17) Variations in QALY had a big impact on ICER (as shown in Figure 2) with the upper quadrant sitting much above the WTP threshold. This should be considered when interpreting the results of this study.<br><br>Discussion<br><br>18) When the authors state that the intervention is cost-effective it is important that they also specifically state what WTP they are considering. The initial statement that both AE and SE from both perspectives were cost-effective is misleading, as the probability of AE being cost-effective was much lower from a healthcare perspective. Conclusions should not be made based on point estimates only (ICERs) but should also consider uncertainty (expressed in acceptability curves) and robustness of the results (sensitivity analysis).<br><br>It is also misleading to base conclusions on negative ICERs as the negative sign could be a result of a less effective intervention (which was not the case here). Some health economists even advise that ICERs should not be calculated in these cases to avoid confusion.<br><br>Conclusion<br><br>19) As pointed above, the sentence below is misleading: "In conclusion, the present study demonstrated that implementing SE or AE for patients with symptomatic knee OA is a cost-effective alternative compared to usual care within the Norwegian healthcare system."

**REVIEWER**	Cai, Jingheng<br>Sun Yat Sen Univ, Statistics
**REVIEW RETURNED**	23-Feb-2024

**GENERAL COMMENTS**	Given that I am not an expert in the medical field, I only comments on the statistical analysis which is rather simple in the manuscript. Some details need to be presented more clearly.<br><br>1. Student's t-test was employed in the analysis, therefore, it is necessary to examine whether the data is distributed as normal distribution.<br><br>2. Page 10, Sample size. I am not sure whether the authors calculate the sample sizes in this study since I did not find any details.<br><br>3. The sample sizes presented in Page 11 (55 to SE, 56 to AE, and 57 to UC) are different from those presented in Abstract (54, 53, and 54).

VERSION 1 – AUTHOR RESPONSE

**Reviewer #1**<br>*I thank the authors for this work, and I find the paper interesting.*		
My major concerns is around the estimation of cost. The cost components in Table 1 does not seem to capture all relevant cost. It is good for readers to have a comprehensive view of the components for replicability of this works elsewhere. What about the cost exercise at home? Purchase of exercise equipment? Co st of	Thank you for this valid comment. Unfortunately, we were not able to include all relevant costs related to healthcare utilization and productivity loss in this study. For sure that is a limitation. Yet, to the best of our knowledge, this is often the case in these types of studies. To promote transparency regarding our cost estimation we have provided an overview of all included cost components, along with the unit price, in Table 1. Additionally, we have clarified in the method and discussion section that the following costs were not estimated: non-healthcare costs related to provision of healthcare, costs related to medication and diagnostic imaging, and costs related to productivity loss related to presenteeism and unpaid work (page 9 line 10-12; page 15, line 25; page 16, line 1 and 7-10).<br><br>We agree that your comment about costs related to equipment should be addressed. Thus, we have incorporated this information in the method section.	Page 9, line 13-14<br>*“..(such as transportation and exercise equipment) were not estimated.”*

registration/attendance of gymnasium for home exercise.<br><br>What major cost components constitutes the "usual care"? Readers need to know because it is unclear at the moment. Medications taken e.g., NSAIDS taken? And Duration?<br><br>Given that the QALY are similar, and 'cost' is the major driver of the cost saving estimated in the study. The authors need to be clear and spot-on on the cost components.		
Why should transportation costs be taken off? This cost is required to measure the societal cost of the intervention.	Unfortunately, there was no available data on transportation costs in the dataset. Thus, we did not actively exclude these costs; rather, we simply lacked this information. We acknowledge that this is a limitation. Yet, to the best of our knowledge, this cost component is not required to calculate direct cost. We calculated direct cost based on all available information within the data material and have tried to be transparent in our manuscript.	No action done.
The authors need to be explicit on what constitutes the cost components of each intervention.	Thank you for this valuable comment. We acknowledge and apologize for not clearly including this information in the manuscript. The cost component of the SE and AE intervention was physiotheray consultations related to the two interventions. The mean (SD) intervention cost per patient was €190 (€433) and €201 (€580) for the SE and the AE intervention, respectively. We have now addedd this information to the result section.	Page 12, line 9-12<br><br>*"Mean (SD) intervention cost per patient was €190 (€433) and €201 (€580) for the SE and the AE intervention, respectively. Costs*

		related to healthcare utilization (including physiotherapist consultations related to the study interventions) and productivity loss for the one-year of follow up are shown in Table 4."
It will be informative to see the healthcare utilisation for each intervention/comparator to know how the different groups utilised healthcare.	To the best of our knowledge, this information is provided in Table A2, A3 and A4 in the additional file 3 which shows healthcare utilization (including physiotherapist consultations related to the study interventions) and productivity loss throughout one-year of follow-up for the three groups separately.	No action done.
**Reviewer #2**<br>*Thank you very much for the opportunity to review this manuscript. Cost-effectiveness investigations are relatively rare in this field, so this manuscript brings an important contribution to the field. Overall, the economic evaluation was well-performed and reported however in several aspects it lacks details. Additionally, the nuances in the interpretation of results need to be considered and clearly stated. I have added suggestions below that aim to improve the clarity of the manuscript.*		
Abstract: I would suggest that you include the ICERs with Bootstrapped confidence intervals.	Thank you for your input. During the initial stages of drafting, we actually discussed whether to include the ICERs into the abstract. However, as you have noted below, interpreting ICERs can pose challenges, especially negative ICERs which might represent reduced cost and positive effects indicating a win–win situation or increased costs and negative effects indicating a lose–lose situation. Consequently, we decided to omit the ICERs from the abstract. Instead, we adhered to the recommendations outlined by van Dongen et al. (2014) and favored to present the results obtained from the Cost-Effectiveness Acceptability Curves (CEACs), as the CEAC provides a summary measure of the joint uncertainty of costs and effects. Based on these considerations, we hope it is acceptable that we do not include the ICERs in the abstract.	No action done.

	(Ref: van Dongen et al. 2014: Trial-Based Economic Evaluations in Occupational Health Principles, Methods, and Recommendations)	
Abstract: It would be helpful to state that the main timepoint for the analysis was 12 months.	Our apologies if we do not understand you question. We agree, stating the timepoint for the analysis is important, thus several places within the abstract we have stated that the timepoint of the analysis is one-year (in the title, in the description of the outcome measure and in the conclusion). We have used the term "one-year" instead of "12 months" to maintain consistent wording throughout the manuscript and we hope it is okay. Nevertheless, we acknowledge that this is a subjective matter, likely contingent on individual preferences and accustomed practices.<br><br>To enhance clarity regarding the analysis timepoint, we have now included it in the result section of the abstract as well.	Page 2, line 15-23<br><br>*"From a one-year societal perspective, mean cost per patient was €7954, €8101, and €17 398 in the strength exercise, a erobic exercise, and usual care group, respectively. From a one-year healthcare perspec tive, mean cost per patient was €848, €2003, and €1654 in the strength exercise, a erobic exercise, and usual care group, respectively. Mean differences in costs significantly favoured strength exercise a nd aerobic exercise from a one-year societal perspective, and strength exercise fr om a one-year healthcare perspective. There were no significant differences in mean QALYs between groups. From a one-year societal perspective, at a willingness-to-pay threshold of €27 500, the probability of strength exercise o r aerobic exercise being cost-effective was ≥ 98%. From a*

		one-year healthcare perspective, the probability of strength exercise o r aerobic exercise being cost-effective was ≥ 97% and ≥ 76%, respectively."
Methods: The authors described that healthcare utilization and productivity loss data were collected at 6 and 9 months. If the time horizon for this study was 12 months, how was healthcare utilisation collected for the last 3 months of the trial (month 10 to 12)?	Thank you for your comment and we apologize for not having included this information clearly in the manuscript. Data on healthcare utilization (except surgery) and productivity loss were collected by questionnaires at 14 weeks (post intervention) and 12 months after inclusion. In addition healthcare utilization and productivity loss were recorded by phone interviews at 6 and 9 months after inclusion (to avoid a recall period > 3 months). Data on knee replacement surgery was collected from registry data.<br><br>We have now made some small changes in the method section to clarify this for the readers. Thank you.	Page 7, line 8-9<br><br>*"Follow-up questionnaires covering healthcare utilization, producti vity loss (sick leave, work assessment allowance and disability benefits) and health state were sent at 14 weeks (post intervention) and 12 months after inclusion."*
Methods: Whic h instrument was used to collect healthcare utilization and productivity loss? If a validated questionnaire was not used (which seems to be the case), I would suggest that the authors include the instruments as supplement materials.	Healthcare utilization (except surgery) and productivity loss was self-reported and not measured with a standardised validated method. We have included this information in the discussion section as a limitation of the study (page 15, line 24-25; page 16, line 4-6).<br><br>We have now incorporated the questions used to measure healthcare utilization and productivity loss into the supplementary materials and made references to this within the manuscript. Thank you.	Figure A1 in the additional file 1<br><br>Page 8, line 3 and 6<br><br>*"… was self-reported and included consultations to healthcare professionals (type and frequency) (Figure A1 in the additional file 1)."*<br><br>*"... was self-reported and included number of calendar days with*

		sick leave, work assessment allowance, and disability benefits (Figure A1 in the additional file 1)."
Methods: Why did the authors restrict healthcare utilisation to utilization due to knee OA? One limitation of this approach is that the intervention might have benefits or harms that expand beyond what would have been captured by "healthcare utilisation due to knee". It would be helpful to add this point to the discussion.	Thank you for you comment. In the datamaterial, we "only" had data on healthcare utilization related to knee OA. Therefore, it was never feasible to estimate costs relate to healthcare utilization in general. In our opinion, this does not represent a distinct limitation that needs to be adressed and prioritised in the discussion section.<br>We agree, one could argue that it is a limitation that healthcare utilization is restricted to utilization due to knee OA, as the intervention might have influenced healthcare utilization in general, and not just healthcare utilization related to knee OA. Yet, one could also argue the opposite, that it is a strenght, as measuring healthcare utilization in general would encompass costs not influenced by the intervention, thus not relevant in this context. To the best of our knowledge, this is an ongoing point of discussion in the field of health economics without clear consesnus. Considering the above, we decided not to make changes to the discussion section in relation to this matter. However, we are open to include this point it the reviewer think it enhance the strength of the paper.	No action done.
Methods: Was the person conducting the interviews blinded to group allocation? The authors state that group allocation was concealed for the assessors, but it would be helpful to make it clearer if this also applies to the people conducting the interviews to	Thank you for your comment and we apologize for not having included this information clearly in the manuscript. The person conducting the phone interviews was blinded to group allocation. We have now incorporated this information more clearly in the method section. Thank you.	Page 6, line 12<br><br>*"Group allocation was concealed for the researchers and the self-reported data assessors."*

collect information on healthcare utilisation and productivity.		
Methods: The recall bias is an important limitation of this study and should be discussed.	Thank you for you comment. We agree. The risk of recall bias is important to mention in the discussion. In the manuscript we have made this comment: *"A third limitation is the fact that we expect to have somewhat underestimated total healthcare utilization and related costs. Self-reports tend to underestimate the true value of healthcare utilization due to potential recall bias".*	No action done.
Methods: What was the location for the exercise sessions? Were participants in the AE group given a stationary bike to take home? Or did they have to travel to a gym? Were physiotherapists the only professionals supervising the exercise classes? Did participants exercise in groups? Or individually? Although the authors cited a reference where the details of the intervention have been described, it would be helpful to have this information described as they have an impact on	Thank you for this valuable comment. During the early writing phase, we deliberated on whether to include a more comprehensive description of the intervention, and decided to make the description as short as possible. However, considering your feedback, we acknowledge the merit in including additional information.<br><br>A minimum of two exercise sessions per week were supervised, while the third session could be perfomed unsupervised at home. The location for the supervised strength- and aerobic exercise sessions were physiotherapy clinics located near the participant's homes. Physiotherapist were the only professionals supervising the exercise interventions. All participants exercised individually. We have now incorporated this information in the method section. Thank you.	Page 6, line 25<br>Page 7, line 1-3<br><br>*"A minimum of two exercise sessions per week were supervised by a physiotherapist, while the third session could be performed unsupervised at home. All supervised SE and AE exercise sessions took place at physiotherapy clinics situated near the participant's homes. All participants exercised individually."*

costs.		
Methods: How were intervention costs calculated? It seems that only physiotherapy staff costs were captured as part of the resource utilization data collection. Were there other costs involved with implementing the intervention in terms of equipment/materials, travel time, rental (location)? These need to be considered and reported.	The intervention costs solely captured costs related to the physiotherapy consultations. There were no additional costs involved with implementing the intervention in terms of equipment and rental of the location.<br>While the physiotherapy clinics varied in the equipment, they all had equipment for leg-press and leg-extention exercises, as well as stationary bicycles, consistent with common practice in Norwegian physiotherapy. Therefore, there were no extra intervention costs related to this aspect. Regarding transportation, as mentioned above, there was no available data within the dataset, making it impossible to calculate this cost. In the method section we have incorporated this information.	Page 9, line 13-14<br>*"Non-healthcare costs related to provision of healthcare (such as transportation and exercise equipment) were not estimated.."*
Method: I would suggest that authors discuss the limitations of valuing productivity according to employment rate, particularly considering people who might not be at paid work.	Thank you for a valuable comment. In the discussion section we have tried to highlight this limitation: *"Also, we lack data on productivity loss related to reduced productivity while at paid work (presenteeism) and unpaid work, thus also expect to have somewhat underestimate the true value of productivity loss*". However, taking your comment into consideration, we recognize that we can elaborate on this point and clearify that this limitation likely holds the greatest significance for people not in paid work. We have now revised the sentence.	Page 16, line 18<br><br>*"Also, we lack data on productivity loss related to reduced productivity while at paid work (presenteeism) and unpaid work, thus also expect to have somewhat underestimate the true value of productivity loss, especially among people not in paid work."*
Methods: Was the identification of outliers for the sensitivity analysis conducted	Yes, the identification of outliers for the sensitivity analysis was blinded for group allocation. Outliers were identified when all participants were merged into one group.	No action done.

blinded to group? As a specific criterion was not set for defining an outlier it is possible that group allocation knowledge could impact on the judgement.		
Methods: It is usually recommended that conversion of currencies be conducted using purchasing power parity conversion factors.	Our apologies if we do not understand you question. We agree. It is recommended to conduct conversion of currencies, by using a currency converter that accounts for differences in price levels between countries, to enable comparison between studies. To the best of our knowledge, when publishing studies on costs from Norway, it is common practice to convert Norwegian Krone (NOK) into euros. Therefore, we also intended to do so in this study. As far as we know, "purchasing power parity coversion factors" do not facilitate conversion to euros. Thus, we, as others, utilized another credible source (the Norwegian Bank of Norway) for currency conversion into euros (€1 = NOK10). We hope it is okay that we refrain from including a comment on this within this manuscript.	No action done.
Results: It would be helpful if bootstrapped 95% CI intervals could be reported for the ICER measures.	Thank you for your input. We agree, it is important representing the uncertainty surrounding the ICERs. Nevertheless, as a ratio measure, estimating 95% CIs around ICERs is not straightforward and, more importantly, 95% CIs around ICERs suffer from the same interpretation problem as the ICERs (as mentioned above). Consequently, we have not included the 95% CIs of the ICERs. Instead, we have followed the recommendations outlined by van Dongen et al. (2014) and depicted the uncertainty surrounding the ICERs through bootstrapped cost-effectiveness planes (CE-planes) and CEACs. Based on these considerations, we hope it is acceptable not to include 95% CI for the ICERs.<br><br>*(Ref: van Dongen et al. 2014: Trial-Based Economic Evaluations in Occupational Health Principles, Methods, and Recommendations)*	No action done.
Results: Intervention costs should be reported separately. A detailed breakdown of	Thank you for your comment. We acknowledge and apologize for not clearly including this information in the manuscript. The intervention cost was costs related to physiotherapy consultations during the intervention period. The mean (SD) intervention cost per patient was €190 (€433) and €201 (€580) for the SE and the AE intervention,	Page 12, line 9-12<br><br>*"Mean (SD) intervention cost per patient was €190 (€433) and €201 (€580) for the*

the intervention costs would be very helpful to inform people who are planning to implement a similar program in the future.	respectively. We have now added this information to the result section.	*SE and the AE intervention, respectively. Costs related to healthcare utilization (including physiotherapist consultations related to the study interventions) and productivity loss for the one-year of follow-up are shown in Table 4."*
Results: Figure 1: it would be helpful to indicate the selected WTP threshold in the acceptability curves in Figure 1 (perhaps with a dotted line?).	Thank you for a valuable comment. We agree and have added the WTP threshold in the acceptability curves in Figure 1 with a dotted line.	Figure 1.
Results: Fig 1A. The acceptability curve indicates that the intervention has close to a 100% probability of being cost-effective considering various WTP thresholds. However, looking at the cost-effectiveness plane there are quite a few points where the intervention was more costly (north quadrant) and more (NE) or	Thank you for your comment. We have now reviewed the results and can confirm their accuracy. As you point out, in Fig 1a, the CEAC indicates close to a 100% probability of cost-effectiveness, while the CE-plane also shows some points in the NE, NW, and SW quadrant. The explanation for the flat curve in the CEAC is, to the best of our knowledge, likely due to differences in the axis scales between the CE-plane and the CEAC, as well as the small differencs in QALYs. Presumably, shrinking the x-axis of the CEAC would result in an upward curve.<br><br>Regarding Fig 1b, we anticipate the small decending of the curve in the CEAC to be related to the fact that we in the CE-plane also see some points located in the SE quadrant.	No action done.

less effective (NW) than usual care. These results don't seem to match what is presented in the acceptability curve. I would only expect a flat line in a situation where all points are located in the southeast quadrant. Similarly, Fig 1B shows that the probably of an intervention being cost-effective decreases as WTP increases which is contra-intuitive. I would suggest that authors check these results.		
Results: Variations in QALY had a big impact on ICER (as shown in Figure 2) with the upper quadrant sitting much above the WTP threshold. This should be considered when interpreting the results of this study.	Thank you for you comment. As mentioned, altering QALYs had a notable impact on the ICER values (due to very small group differences circling around zero). In the last paragraph of the result section, we made a comment regarding this: *"Altering QALYs, however, had a significantly effect on the ICERs and led to some ICERs above a WTP threshold of €27 500 per QALY gained."* Furthermore, when summarising the results in the discussion section, we emphasized this point: *"Although, results were sensitive to different estimates of QALYs (due to very small group differences circling around zero)..".* Considering your comment, we acknowledge the importance of also mentioning this aspect in the conclusion, and have now revised the text.	Page 17, line 12-13<br><br>*"Yet, the results were sensitive to different estimates of QALYs (due to very small group differences)."*
Discussion: When the authors state	We agree on the importance of emphasizing the WTP threshold when asserting the cost-effectiveness of the	Page 14, line 12

that the intervention is cost-effective it is important that they also specifically state what WTP they are considering. The initial statement that both AE and SE from both perspectives were cost-effective is misleading, as the probability of AE being cost-effective was much lower from a healthcare perspective. Conclusions should not be made based on point estimates only (ICERs) but should also consider uncertainty (expressed in acceptability curves) and robustness of the results (sensitivity analysis).	interventions. We have now included the specific WTP threshold being utilized. Thank you.<br><br>We also agree on the importance of not solely relying on point estimates (ICERs). Therefore, in both the results, discussion and conclusion sections we have emphasized findings from the CE-planes, the CEAC, and the sensitivity analysis.	*"From a societal and healthcare perspective both SE and AE were found to be cost-effective at a WTP threshold of €27 500."*
Discussion: It is also misleading to base conclusions on negative ICERs as the negative sign could be a result of a less effective intervention (which was not the case here). Some health	Thank you for your comment. We are aware that there are different opinions regarding drawing conclusions based on negative ICER values. Therefore, in the conclusion section, we have chosen not to adress the negative ICER values. Instead, we have simply stated that the ICER values were below the WTP threshold of €27 500, indicating that SE and AE are cost-effective alternatives compared to usual.	No action done.

economists even advise that ICERs should not be calculated in these cases to avoid confusion.		
Conclusion: As pointed above, the sentence below is misleading: "In conclusion, the present study demonstrated that implementing SE or AE for patients with symptomatic knee OA is a cost-effective alternative compared to usual care within the Norwegian healthcare system."	As mentioned above, we agree on the importance of emphasizing the WTP threshold when asserting the cost-effectiveness of the interventions. We have now included the specific WTP threshold being utilized also in the conclusion section. Thank you.	Page 17, line 11<br><br>*"In conclusion, the present study demonstrated that implementing SE or AE for patients with symptomatic knee OA is a cost-effective alternative compared to usual care within the Norwegian healthcare system, at a WTP threshold of €27 500."*
**Reviewer #3**<br>*Given that I am not an expert in the medical field, I only comments on the statistical analysis which is rather simple in the manuscript. Some details need to be presented more clearly.*		
Student's t-test was employed in the analysis, therefore, it is necessary to examine whether the data is distributed as normal distribution.	Thank you for your comment. The cost data within this study were skewed (as expected and commonly observed in such studies). Skewed cost distribution complicates cost data analysis by violating the assumptions of standard statistical tests. Typically, skewed data are summarized using the median.<br>However, this approach is deemed inappropriate for cost data, as decision-makers need to be able to estimate the total cost of implementing a new intervention. Consequently, the mean value is generally considered the most informative measure for describing cost data. With regards to methods used to compare cost data between study arms, various methods are currently being used, including standard nonparametric tests and parametric tests such as the Student's t test. However, it has been argued that nonparametric tests are inappropriate for cost data analysis as they compare the distribution of the	No action done.

	data rather than the means. Therefore, we decided to utilize the Student's t test when comparing cost data between study arms. Based on the above, we hope you agree that we do not make any changes to the manuscript in relation to this matter.<br><br>Note. We actually also conducted these analyses using a non-parametric method (the Mann-Whitney U test), which provided similar results.	
Page 10, Sample size. I am not sure whether the authors calculate the sample sizes in this study since I did not find any details.	Thank you for you comment. As mentioned in the method section, this study contains secondary analysis embedded in a RCT. Sample size calculation related to the primary aim of the RCT has been provided in the primary result paper *(Øiestad et al. 2023. The efficacy of strength or aerobic exercise on quality of life and knee function in patients with knee osteoarthritis. A multi-arm randomized controlled trial with 1-year follow-up*). There has been no sample size calculation for this study. In the discission section, we have mentioned this as a limitation of the study: *"The main limitation of the present study is that it was powered on the primary outcome of the three-arm RCT, not on the cost-effectiveness outcome. However, this is common practice in trial-based economic evaluations, due to the right-skewed distribution of cost data and the fact that an extremely large sample size is required to detect relevant cost differences, which in turn can both infeasible and/or unethical. To address this limitation, as recommended, bootstrapped estimates were used for result interpretation."*	No action done.
The sample sizes presented in Page 11 (55 to SE, 56 to AE, and 57 to UC) are different from those presented in Abstract (54, 53, and 54).	Thank you for your comment and we appoligies for the confusion. A total of 168 participants were randomized into the three groups: 55 to SE, 56 to AE, and 57 to UC. Seven participants withdrew their informed consent, leaving 161 participants for the ITT analyses (54 in SE, 53 in AE and 54 in UC). To improve clarity regarding the sample size in the result section, we have made some minor adjustments to the text in this section.	Page 11, line 14-15<br><br>*"Seven participants (4%) withdrew their informed consent (due to lack of time or not attending tests), leaving 161 (96%) participants for the ITT analyses (54 to SE, 53 to AE, and 54 to UC)."*

VERSION 2 – REVIEW

**REVIEWER**	Cai, Jingheng<br>Sun Yat Sen Univ, Statistics
**REVIEW RETURNED**	25-Mar-2024

**GENERAL COMMENTS**	I am still not comfortable with the t-test. Since the data is not normal distributed, the t-test is not an appropriate method to conduct the hypothesis testing.

**REVIEWER**	Okafor, Charles<br>The University of Queensland, Centre for Health Services Research
**REVIEW RETURNED**	03-Apr-2024

**GENERAL COMMENTS**	While the authors have addressed most of this reviewer's concerns, there are some comments which were not satisfactorily addressed.<br>The study employed societal and healthcare perspectives in the costing. While the authors have noted they could not collect transportation cost data, using international data or proxy data to represent this cost at least in a sensitivity analysis would be helpful.<br>More importantly, the cost of exercise equipment for home exercise or gym registration are valid and reasonable cost estimates that should be considered. At a minimum, secondary data should be used to value this cost item in a sensitivity analysis. The key idea is to simulate or capture all relevant costs to guide decision-making.<br>At the moment, this reviewer is not confident of the reliability, replicability and validity of the cost estimates of this study.

VERSION 2 – AUTHOR RESPONSE

Reviewer #3: I am still not comfortable with the t-test. Since the data is not normal distributed, the t-test is not an appropriate method to conduct the hypothesis testing. Authors' response: Thank you for your comment. We agree, the skewed data distribution complicates the analysis of cost data, as it violates the assumptions of parametric tests. A standard approach to compare skewed data is to use a non-parametric test. However, non-parametric tests compare data distribution instead of means (which are considered the most informative measure for describing cost data) and are therefore considered to be inappropriate when comparing cost data between study arms (van Dongen et al. 2014). Consequently, we, like others, used the Student's t test when comparing cost data between study arms. Nevertheless, we acknowledge that some might share the same concern as you regarding the appropriateness of using a parametric test on skewed data. Therefore, during the analysis phase, we also conducted these analyses using a non-parametric test (the Mann-Whitney U test), which provided similar results. However, this information was not included in the manuscript. Based on your comment, we have now incorporated the Mann-Whitney U test as a sensitivity analyses, and made some changes to the method and result section. We hope that you find these changes appropriate and that you can support publication of this study. (Ref: van Dongen et al. 2014: Trial-Based Economic Evaluations in Occupational Health Principles, Methods, and Recommendations) Authors' action: Page 11, line 5-7: "To evaluate the appropriateness of analysing groups differences in costs (skewed data) with a parametric test (Student's t-test), a sensitivity analysis was conducted with the MannWhitney U test." Page 13, line 4-5: "The sensitivity analyses

conducted with the Mann-Whitney U test provided similar results in terms of statistical significance." Reviewer #1: While the authors have addressed most of this reviewer's concerns, there are some comments which were not satisfactorily addressed. The study employed societal and healthcare perspectives in the costing. While the authors have noted they could not collect transportation cost data, using international data or proxy data to represent this cost at least in a sensitivity analysis would be helpful. More importantly, the cost of exercise equipment for home exercise or gym registration are valid and reasonable cost estimates that should be considered. At a minimum, secondary data should be used to value this cost item in a sensitivity analysis. The key idea is to simulate or capture all relevant costs to guide decisionmaking. At the moment, this reviewer is not confident of the reliability, replicability and validity of the cost estimates of this study. Authors' response: Thank you for your comment. We agree, the ideal is to capture all relevant costs to guide decision-making. We were not able to include all relevant costs in this study, including costs related to transportation and exercise equipment, which is a limitation. Based on your recommendation and to evaluate the effect of not including transportation costs related to the interventions, we have now added a sensitivity analysis in which we have included an estimated average transportation cost. Transportation costs per patient were estimated by multiplying number of physiotherapy consultations in the intervention period by an estimated average transportation cost of €7 (based on a government-set rate of €0.35 per kilometer for an estimated average round-trip distance of 20 kilometres). No substantial change in point estimates of cost (€) and effect (QALYs) differences were seen when comparing analysis including transportation costs related to the intervention to the main analysis. With regards to costs related to home exercise equipment or gym registration, none of the participants were asked to acquire any form of home exercise equipment or a gym registration. All exercise sessions could be performed at physiotherapy clinics, where the exercise equipment used is standard, and there is no need for a paid "gym registration". Thus, with these interventions, there were supposed to be no extra costs related to home exercise equipment or a gym registration. We registered no questions from the participants related to buying home exercise equipment during the trial. However, it is possible that participants who chose to preform the third weekly exercise session at home have acquired home exercise equipment anyway. Authors' action: Page 10, line 25; Page 11, line 1-5: To evaluate the effect of not including transportation costs related to the interventions, a sensitivity analysis including an estimated average transportation cost was performed. Transportation costs per patient were estimated by multiplying number of physiotherapy consultations in the intervention period by an estimated average transportation cost of €7 (based on a government-set rate of €0.35 per kilometer for an estimated average round-trip distance of 20 kilometres). Page 13, line 10-12: "Furthermore, no substantial change in point estimates of mean cost (€) and effect (QALYs) differences were seen when comparing analysis including transportation costs related to the intervention to the main analysis (Table A6 in the additional file 6)." Table A6 in the additional file 6

VERSION 3 – REVIEW

**REVIEWER**	Okafor, Charles<br>The University of Queensland, Centre for Health Services Research
**REVIEW RETURNED**	05-May-2024

**GENERAL COMMENTS**	None

**REVIEWER**	Cai, Jingheng<br>Sun Yat Sen Univ, Statistics
**REVIEW RETURNED**	30-Apr-2024

**GENERAL COMMENTS**	I have no further comments.
